# Accelerated Navigator for Rapid ∆B_0_ Field Mapping for Real‐Time Shimming and Motion Correction of Human Brain MRI

**DOI:** 10.1002/nbm.70126

**Published:** 2025-09-04

**Authors:** Nutandev Bikkamane Jayadev, Jason Stockmann, Robert Frost, Nicolas Arango, Yulin Chang, André van der Kouwe, Ovidiu C. Andronesi

**Affiliations:** ^1^ Athinoula A. Martinos Center for Biomedical Imaging Massachusetts General Hospital Charlestown Massachusetts USA; ^2^ Siemens Medical Solutions USA Inc. Malvern Pennsylvania USA; ^3^ Department of Radiology Harvard Medical School Boston Massachusetts USA; ^4^ Department of Electrical Engineering and Computer Science Massachusetts Institute of Technology Cambridge Massachusetts USA

**Keywords:** GRAPPA acceleration, magnetic resonance spectroscopic imaging (MRSI), motion correction, multi‐coil shim array, real‐time shimming, spherical harmonic shims, volumetric navigators (vNavs), ΔB₀ field mapping

## Abstract

∆B_0_ shim optimization performed at the beginning of an MR scan is unable to correct for ∆B_0_ field inhomogeneities caused by patient motion or hardware instability during scans. Navigator‐based methods have been demonstrated previously to be effective for motion and shim correction. The purpose of this work was to accelerate volumetric navigators to allow fast acquisition of the parent navigated sequence with short real‐time feedback time and high spatial resolution of the ∆B_0_ field mapping. A GRAPPA‐accelerated 3D dual‐echo EPI vNav was implemented on a 3 T Prisma MRI scanner. Testing was performed on an anthropomorphic head phantom and 11 human participants. vNav‐derived ∆B_0_ field maps with various spatial resolutions were compared to Cartesian‐encoded gold‐standard 3D gradient‐echo ∆B_0_ field mapping. ∆B_0_ shimming was evaluated for the scanner's spherical harmonics shims and a custom‐made AC/DC RF‐receive/∆B_0_‐shim array. The performance of dual‐echo and single‐echo accelerated navigators was compared for tracking and updating ∆B_0_ field maps during motion. Real‐time motion and shim corrections for 2D MRI and 3D MRSI sequences were assessed in vivo with controlled head movement. Up to 8‐fold acceleration of volumetric navigators (vNavs) significantly reduced geometric distortions and signal dropouts near air‐tissue interfaces and metal implants. Acceleration allowed a flexible tradeoff between spatial resolution (2.5–7.5 mm) and acquisition time (242–1302 ms). Notably, accelerated high‐resolution (5 mm) vNav was faster (378 ms) than unaccelerated low‐resolution (7.5 mm) vNav (700 ms) and showed better agreement with 3D‐GRE ∆B_0_ field mapping with 5.5 Hz RMSE, 1 Hz bias, and [−10%, +10%] confidence interval. Accelerated vNavs improved 3D MRSI and 2D MRI in real‐time motion and shim correction applications. Advanced shimming with spherical harmonic and shim array showed superior ΔB_0_ correction, especially with joint shim optimization. GRAPPA‐accelerated vNavs provide fast, robust, and high‐quality ∆B_0_ field mapping and shimming over the whole‐brain. The accelerated vNavs enable rapid correction of ∆B_0_ field inhomogeneities and faster acquisition of the navigated parent sequence. This methodology can be used for real‐time motion and shim correction to enhance data quality in various MRI applications.

## Graphical abstract

Flowchart of the real‐time motion and shim correction, including image acquisition by the accelerated volume navigator, image processing for motion tracking and ∆B_0_ field mapping, and hardware update.

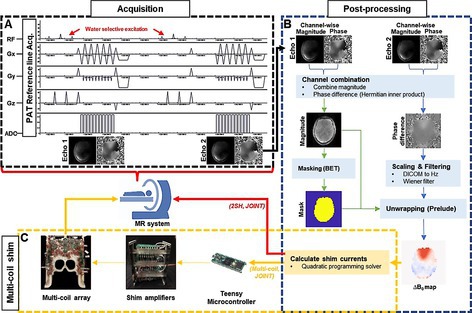

**Abbreviations**
MRI: Magnetic resonance imaging
EPI: Echo planar imaging
vNav: Volumetric navigator
GRAPPA: Generalized auto‐calibrating partially parallel acquisition
GRE: Gradient echo
MRSI: Magnetic resonance spectroscopic imaging
PAT: Parallel Acquisition Technology (Siemens acronym)
TE: Echo time
TR: Repetition time
ACS: Auto‐calibration signal
BET: Brain Extraction Tool
FSL: FMRIB Software Library
PRELUDE: Phase Region Expanding Labeller for Unwrapping Discrete Estimates
2SH: Second‐order spherical harmonic
PACE: Prospective Acquisition Correction (Siemens acronym)
ICE: Image Calculation Environment (Siemens acronym)
RMSE: Root mean squared error
MoCo: Motion correction
NoCo: No correction
MoShCo: Motion and shim correction
GOIA: Gradient offset independent adiabatic
NAA: N‐acetyl‐aspartate
CRLB: Cramer–Rao lower bound
FWHM: Full width at half maximum
SNR: Signal‐to‐noise ratio
ROMEO: Rapid opensource minimum spanning tree algorithm

## 1 Introduction

Advancing magnetic resonance imaging (MRI) methods to counteract the challenges posed by ∆B_0_ field inhomogeneities, especially under the influence of patient motion, is essential for optimal image quality. Traditional shim optimization techniques help improve static ∆B_0_ homogeneity for the subject‐specific susceptibility distribution by acquiring ∆B_0_ field maps and then applying compensating spherical harmonic fields. However, the ∆B_0_ field mapping and shimming are typically executed just before the scan commences and are ineffectual against intra‐scan ∆B_0_ field fluctuations caused by patient movement [1, 2, 3] or by hardware instability [4].

Two primary strategies have emerged to monitor and correct dynamic changes in the ∆B_0_ field [5, 6]: external field probes [7, 8, 9, 10] and navigator‐based methods [11, 12, 13, 14, 15, 16, 17, 18]. The use of external field probes involves adding supplementary hardware to the MRI setup and suffers from limitations in accuracy, primarily due to reliance on extrapolations and data fitting in the anatomical region. On the other hand, navigator‐based methods enable real‐time motion and field estimation by harnessing the MR data itself, representing a more efficient approach without the need for additional hardware.

In our prior work, we successfully leveraged whole‐brain dual‐echo echo planar imaging (EPI)‐based volumetric navigators (vNavs), demonstrating their effectiveness in rapid field measurement and real‐time shim updates for spectroscopic imaging [15, 16]. These studies used a low‐resolution 3D vNav with an 8 mm isotropic resolution, acquisition matrix 32 × 32 × 18, and a total navigator duration of 720 ms. While effective, the navigator's long duration increased the minimum repetition time of the parent sequence, thereby extending the overall acquisition time. Additionally, the low‐resolution (8 mm) navigator voxels were susceptible to ∆B₀ inhomogeneity in brain regions with large variations in susceptibility, leading to signal dropout and potentially affecting the accuracy of ∆B₀ field mapping.

In the current study, we aimed to address the limitations of dual‐echo EPI vNavs by accelerating their acquisition with generalized auto‐calibrating partially parallel acquisition (GRAPPA) [19]. Specifically, we sought to shorten acquisition duration, enhance spatial resolution [20], and reduce geometric distortions to improve the reliability of ∆B₀ field mapping. Accelerated whole‐brain vNav‐based field maps acquired at various spatial resolutions and acceleration factors were compared to high‐quality gradient echo (GRE) reference field maps with 2.5 mm isotropic resolution. Although GRE field maps provide accurate and robust static ∆B₀ measurements, their inherently long acquisition times preclude their use for real‐time applications. Therefore, we first established whether accelerated dual‐echo vNav protocols could achieve accuracy comparable to GRE for static ∆B₀ mapping, while simultaneously enabling rapid, repeated acquisitions suitable for real‐time shim and motion correction.

To assess the relevance of variations in ∆B₀ field mapping across different vNav protocols, we evaluated their performance in downstream shimming tasks using the scanner's first‐ and second‐order spherical harmonic shims, a custom‐made 32‐channel AC/DC shim array, and their combination. Finally, we showcase the application of accelerated vNav for real‐time motion and shim corrections in both 2D MRI and 3D magnetic resonance spectroscopic Imaging (MRSI), highlighting their effectiveness in motion tracking and ∆B₀ field mapping to update localization and shimming during subject motion.

## 2 Methods

### 2.1 vNav Protocols

**FIGURE 1:**
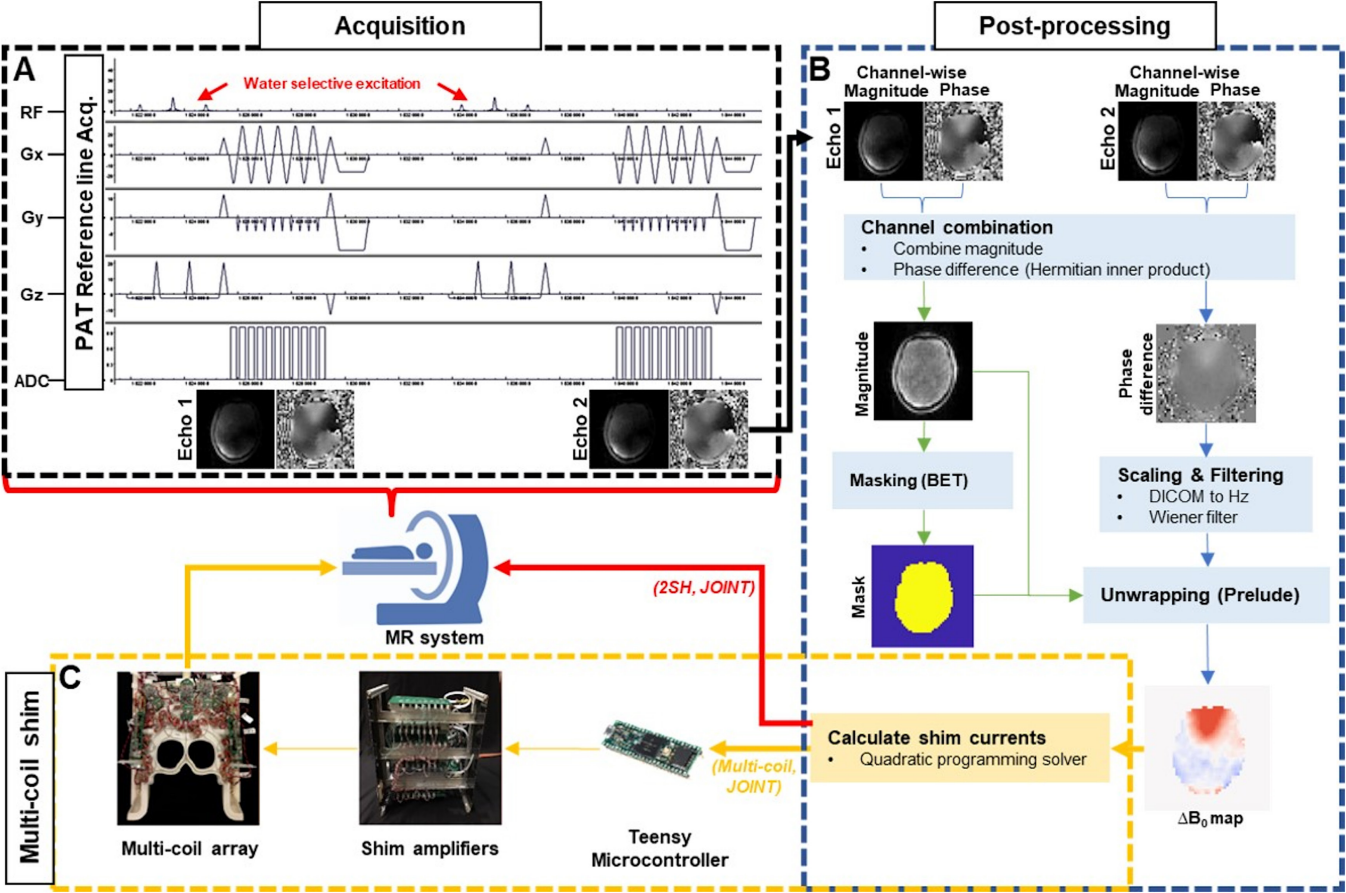
Navigator acquisition and ∆B_0_ field mapping/shimming pipeline. (A) Pulse sequence for GRAPPA dual‐echo EPI volume navigator. (B) Channel‐wise data is acquired and processed for combined phase difference, phase unwrapping, brain masking, computing ∆B_0_ field maps, and calculating shim currents for 2SH, multi‐coil and joint optimization. (C) Uploading of the multi‐coil shim currents to the 32‐channel AC/DC coil.

The GRAPPA‐accelerated 3D dual‐echo EPI vNav was implemented on a 3 T MAGNETOM Prisma MRI scanner (Siemens Healthineers, Erlangen, Germany) equipped with the Siemens 32‐channel phased array head coil and a custom‐made 32‐channel AC/DC shim array coil with associated shim current drivers on each channel [21]. The data acquisition and processing workflow is shown in Figure 1.

**FIGURE 2:**
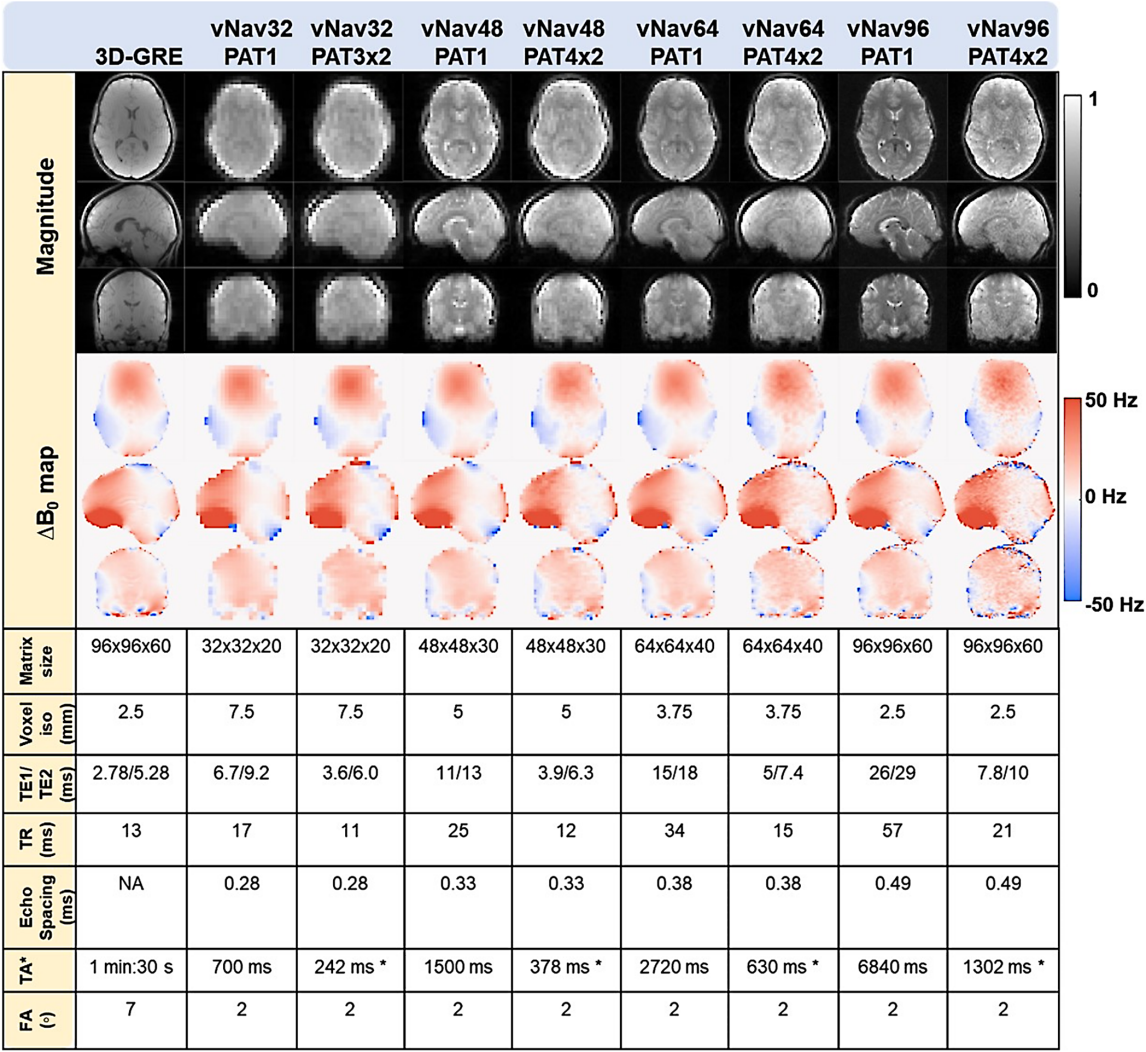
Magnitude and ∆B_0_ field maps from a healthy human volunteer. Gold‐standard 3D‐GRE and EPI‐vNavs (with four resolutions and two accelerations) are shown at their acquired resolution. Respective protocol parameters are tabulated below. The GRAPPA acceleration denotes in‐plane and partition (in‐plane × partition) undersampling factors. The total acquisition time for accelerated sequences (TA*) excludes the duration of the ACS lines.

The acquisition protocol comprised a standard dual‐echo 3D‐GRE sequence followed by a series of eight dual‐echo EPI vNav protocols, encompassing four spatial resolutions and two accelerations. A summary of the imaging protocols and acquisition parameters is provided in Figure 2, where the naming convention denotes the respective matrix sizes and Parallel Acquisition Technology (PAT) acceleration factors. A larger flip angle was used for the 3D‐GRE to optimize signal for robust field mapping, whereas vNavs used smaller flip angles to minimize the effect on the magnetization for the parent sequence. Contrast differences did not affect ∆B₀ mapping accuracy, as identical ΔTE was maintained across protocols. The echo time (TE), repetition time (TR), and echo spacing were configured to the scanner's minimal allowable settings to ensure the fastest possible navigator acquisition. The readout bandwidth remained constant for accelerated and unaccelerated vNavs of the same resolution. The echo time difference was chosen to ~2.4 ms to have in‐phase fat and water signals at 3 T. Additionally, water selective excitation was performed to reduce chemical shift ghosting of the fat signal along the two blipped EPI directions, which provides cleaner magnitude and phase images for head tracking and ∆B_0_ field mapping, respectively. The GRAPPA‐accelerated vNav protocols used GRE‐based auto‐calibration signal (ACS) pre‐scan acquisition. The number of ACS lines for in‐plane and partition directions was set to half the phase encoding matrix size of the respective navigator protocol, and the total time for the ACS lines acquisition was 0.8 s for vNav32, 1.8 s for vNav48, 3.1 s for vNav64, and 7.1 s for vNav94. Note that the total acquisition time reported in Figure 2 for the accelerated vNav protocols excludes the time spent on ACS since ACS needs to be acquired only once at the beginning of a navigated sequence.

### 2.2 Validation Experiments

#### 2.2.1 ∆B_0_ Field Mapping

Protocol validation was performed on an anthropomorphic head phantom [22] and human participants. The participant pool consisted of 11 individuals, including five healthy controls (3M/2F 25–30 years) and six glioma patients (3M/3F, 28–54 years). All participants provided written informed consent, and the study was approved by the local Institutional Review Board. Magnitude and phase images were acquired for all the protocols listed in Figure 2 in a neutral head position without any optimization of the ∆B_0_ field, i.e., using the “tune‐up” ∆B_0_ shim performed at the time of scanner installation/maintenance to ensure the same starting conditions in all experiments.

The data was processed on an offline workstation. Channel combined magnitude images from the first echo were obtained by the sum of squares method. The Brain Extraction Tool (BET) of FMRIB Software Library (FSL) [23] was used to generate a brain mask from magnitude images. The combined phase difference maps were calculated using the Hermitian inner product across channels to remove the background coil phase from each channel [24] and avoid phase singularities in the coil combined phase image. The phase difference from all RF channels was then combined using a simple average. The combined phase difference images were transformed to ∆B_0_ field maps in the following steps: (1) phase difference maps were unwrapped using the FSL Phase Region Expanding Labeller for Unwrapping Discrete Estimates (PRELUDE) tool [25]; (2) the unwrapped phase difference was divided by the echo time difference to convert it into Hz; (3) a Wiener filter was applied to capture the smooth spatial pattern of the ∆B_0_ field; and (4) the brain mask was applied to the phase difference maps to generate ∆B_0_ field maps.

#### 2.2.2 ∆B_0_ Field Shimming

Data was acquired from a phantom and a human brain with the tune‐up shims for 3D‐GRE and vNav sequences using an AC/DC shim array. Baseline ∆B_0_ field maps were obtained for each protocol using the workflow described in the previous section. The ∆B_0_ field maps were then exported and processed offline in MATLAB, where they were used as input in a quadratic program constrained shim optimization solver (MATLAB quadprog), as previously described [21, 26, 27, 28] to perform the following ∆B_0_ shimming experiments: (1) scanner's linear and second‐order spherical harmonic (2SH) shimming, (2) 32‐channel AC/DC multi‐coil shimming, and (3) combined (joint) 2SH + AC/DC shimming. The optimized shim currents were uploaded to the scanner and shim amplifiers, and the shimmed ∆B_0_ field was measured with the 3D‐GRE sequence. The effect of head orientation on ∆B_0_ field mapping was also assessed for three head positions (neutral, right rotation, left rotation).

#### 2.2.3 Real‐Time Motion Correction and ∆B_0_ Shimming

The accelerated vNav was implemented in two “parent” sequences, 2D EPI and 3D MRSI, to assess real‐time motion correction and ∆B_0_ shimming. For each TR, head pose (position and orientation) changes were computed online by co‐registering the magnitude image of the latest vNav to the reference vNav acquired in the first TR using prospective acquisition correction (PACE). This process provided translation and rotation estimates to update localization in real‐time. Pose corrections were applied prospectively to the next parent acquisition and the next vNav acquisition. Prospective correction of the vNav ensures that each vNav motion estimate represents the difference in pose from the last TR. This strategy minimizes the magnitude of each registration, which is beneficial for registration accuracy and allows the pose estimation to be refined over multiple TRs. vNav‐based ∆B_0_ field maps were used to calculate additional shim coefficients required to maintain field homogeneity.

##### 2.2.3.1 Phantom Experiments

An anthropomorphic head phantom was used to validate the performance of the vNav‐based motion correction and ∆B_0_ shimming system. Data were acquired using four vNav protocols (vNav32PAT1, vNav32PAT3x2, vNav48PAT1, and vNav48PAT4x2) interleaved with a 2D EPI parent sequence under four conditions: (i) static, (ii) motion with NoCo (no correction), (iii) motion with MoCo (motion correction), and (iv) motion with MoShCo (motion and shim correction). Scans were performed using a standard 20‐channel head–neck coil, with TE = 30 ms, TR = 4500 ms, FOV = 240 × 240 × 100 mm^3^, matrix 96 × 96 × 35, flip angle = 90°, bandwidth = 2367 Hz/pixel, and measurements = 30. Motion was simulated manually by physically moving the phantom during the interleaved measurements at designated intervals. Specifically, the phantom was rotated by ~15° over five measurements and later translated by ~10 mm over two measurements along the *z*‐direction.

For real‐time shim correction, only the frequency and the linear shim terms (first‐order spherical harmonics) of the standard scanner shim hardware were updated. The first‐order shims are shared with the imaging gradients and have the same controller; hence they can be specified on the same timing raster as the gradients, i.e., 10 ms. Eddy current induced delays ~10 ms, which affect the realization of gradient changes, are compensated for imaging gradients, but may not be for shimming, which has settling times less than 1 ms. Second‐order shims were excluded due to their long settling durations (500–3000 ms), which are incompatible with the TR of the EPI parent sequence. The real‐time feedback delay time was 100 ms, which included all calculations performed online in the Siemens Image Calculation Environment (ICE) platform, including image reconstruction, motion estimates (translation, rotation), slice update, ∆B_0_ field map generation, and the scanner's frequency and first‐order spherical harmonic shim update.

##### 2.2.3.2 In Vivo Experiments

The results obtained by 2D EPI sequence in the phantom experiment served as the rationale to select the vNav48PAT4x2 protocol for subsequent in vivo studies. Accelerated vNav with 5 mm^3^ isotropic resolution and 378 ms duration (vNav48PAT4x2) was interleaved with a high‐resolution 2D EPI sequence (parent‐MRI) to assess prospective slice pose correction and ∆B_0_ mapping (no real‐time shim correction) and a slab‐selective 3D MRSI sequence (parent‐MRSI) to assess prospective slice pose correction and prospective higher‐order shimming with the AC/DC shim array.

Measurements for the 2D EPI parent sequence were performed on a healthy volunteer with a standard 32‐channel head coil, with TE = 30 ms, TR = 3500 ms, FOV = 240 × 240 × 100 mm^3^, matrix 96 × 96 × 35, flip angle = 90°, bandwidth = 2367 Hz/pixel, GRAPPA acceleration = 2, and measurements = 15. The subject was instructed to perform controlled head movements during the scan, which included leftward head rotations up to approximately ~6° and ~4 mm translation. Visible markers were placed to help the subject assume the reference and final positions. Data were acquired for two conditions: (i) motion with real‐time motion correction (MoCo) and (ii) motion with no correction (NoCo).

Real‐time motion correction was performed using PACE registration of the navigator's magnitude image to a reference vNav from initial TR. The estimated head pose (translation and rotations) was applied prospectively to both the next parent and the subsequent navigator; hence, each vNav captured incremental motion from the previous TR. All online computations, including GRAPPA reconstruction, motion estimation, and slice update, were executed in the Siemens ICE with a feedback delay of 100 ms.

To demonstrate improvements with real‐time ∆B_0_ shimming for 3D MRSI, the data were acquired using a navigated adiabatic spin‐echo spiral encoded pulse sequence [29] and the 32‐channel AC/DC (RF‐receive/∆B_0_‐shim) coil on a healthy subject in 4:08 min with TR = 1.8 s, TE = 97 ms, FOV = 220 × 220 × 73 mm^3^, matrix 30 × 30 × 10, isotropic voxel 7.3 mm^3^, spectral window 1350 Hz, and 5 ms × 20 kHz GOIA pulses for slab selection. The reconstructed MRSI data were fitted with LCModel software [30] (version 6.3‐1R) using a simulated basis. MRSI was acquired under four conditions: (i) static, (ii) motion with MoCo (motion correction), (iii) motion with MoShCo (motion and shim correction), and (iv) motion with NoCo (no correction). During motion scans, the subject consistently reproduced rotations (approximately 5–7°), chin‐up/down nodding (approximately 3–5°), and translations (approximately 5 mm). The range of head motion was as large as allowed by the anatomical constraints, the size of the receive helmet, and the padding. The accelerated vNav was used to update MRSI localization (slab excitation and FOV encoding) and the ∆B_0_ shimming of the multi‐coil AC/DC shim array.

For each TR, pose changes were computed online using PACE to update slice position, FOV, and shims as previously described. For the real‐time shim correction, the AC/DC shim currents were calculated off‐line in MATLAB using a linearly constrained quadratic optimization method as previously described [21, 26, 27, 28]. A feedback delay time of 500 ms was used to update the shim array, which included image reconstruction in ICE, motion estimate, and localization update (translation, rotation), off‐line transfer of ∆B_0_ field maps, brain masking, MATLAB shim calculations, and sending the shim currents to the AC/DC of shim amplifiers. Note that the settling time for the shim array is much faster, between 1 and 2 ms, and the major contribution to the duration of feedback delay for the AC/DC update is given by the off‐line data transfer, brain masking, and MATLAB shim optimization.

#### 2.2.4 Evaluation of Single‐Echo Navigator for ∆B_0_ Field Tracking and Shim Update

An additional phantom experiment was performed to evaluate the effectiveness of single‐echo navigators for tracking ∆B_0_ field changes over time. An anthropomorphic head phantom was scanned under both static and motion conditions using a 2D‐EPI parent sequence interleaved with vNav48PAT1 and vNav48PAT4x2 dual‐echo navigator protocols. MoCo was active in all acquisitions. During the motion condition, the phantom was physically moved across five consecutive measurements, simulating a chin‐up motion. The motion was corrected by the navigator in real time using the first echo magnitude images. The ∆B_0_ field map was not updated in real time purposely to verify the performance of the single‐echo navigator under the worst‐case scenario, where the un‐updated ∆B_0_ field is more challenging to map in the moved head position compared to the initial head position. The real‐time shim update was verified retrospectively by simulating shim currents and the updated ∆B_0_ field maps.

Data were acquired using a standard 20‐channel head/neck coil with the following parameters: TE = 30 ms, TR = 8000 ms, FOV = 240 × 240 × 100 mm^3^, matrix 96 × 96 × 35, flip angle = 90°, bandwidth = 2367 Hz/pixel, and measurements = 30. The dual‐echo field maps were computed for each measurement using the Hermitian inner product of the complex data from the two echoes, followed by phase unwrapping using the rapid open‐source minimum spanning tree algorithm (ROMEO) [31] and brain masking.

To emulate single‐echo vNav field tracking, the approach described in Dymerska et al. [32] was implemented in MATLAB. Channel‐wise reference phase offsets and reference ∆B_0_ field maps were calculated using the initial dual‐echo measurement (measurement number 3). The channel‐wise reference phase offsets were subtracted from the first echo phase images of all subsequent measurements to estimate the dynamic phase shift during static and moved conditions. The resulting phase maps were unwrapped using ROMEO, masked, and converted into ∆B_0_ field maps in Hz.

Field maps from both dual‐echo and single‐echo approaches were input into a quadratic programming solver to estimate frequency and first‐order spherical harmonic shim coefficients across all measurements under both static and motion conditions. The voxel‐wise mean volume ∆B_0_ difference for single‐echo and dual‐echo maps was computed for each measurement with respect to the reference measurement to quantify the accuracy of the single‐echo approach for dynamic ∆B_0_ tracking.

### 2.3 Statistical Analysis

To compare ∆B_0_ field maps of different spatial resolutions in voxel‐by‐voxel analysis, the lower resolution vNav field maps were interpolated up to the resolution of the 3D‐GRE field maps. For quantitative analysis, the brain mask generated from 3D‐GRE magnitude images was used across all the interpolated ∆B_0_ field maps.

Whole‐brain histograms and standard deviation metrics were used to assess the distribution and variability in each individual protocol. The voxel‐wise root mean squared error (RMSE) was calculated to quantify the ∆B_0_ field map differences between the vNavs and gold‐standard GRE methods. Bland–Altman analysis was used to identify systematic bias and the limits of agreement.

## 3 Results

**FIGURE 3:**
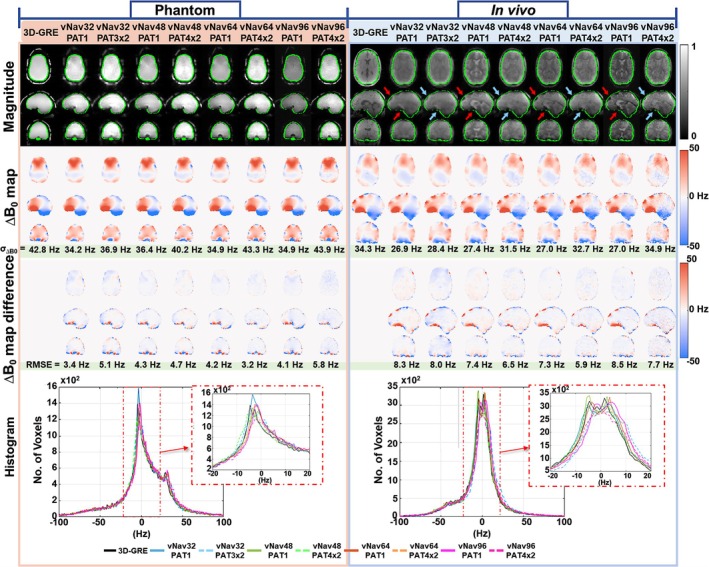
Comparison of magnitude and ∆B_0_ field maps in an anthropomorphic head phantom and a brain tumor patient with metal implants. EPI‐vNavs (with four resolutions and two accelerations) are compared against the gold‐standard 3D‐GRE. The BET brain masks are overlayed on the magnitude images (in green). The low‐resolution vNav images are interpolated to match the 3D‐GRE resolution for statistical comparisons. Corresponding standard deviation for the whole brain volume are shown. ∆B_0_ map difference, RMSE and histograms for vNavs are shown in comparison to 3D‐GRE. All ∆B_0_ field maps are measured using the scanner tune‐up shims. The red arrows indicate areas of signal dropout in the unaccelerated vNav, and the blue arrows indicate signal recovery in the same areas with the accelerated vNav.

Figure 2 provides a visual comparison of magnitude images and ∆B_0_ field maps across different resolutions and accelerations for EPI vNavs, contrasted against the benchmark gold‐standard of 3D‐GRE. The magnitude images show the typical T1 weighted contrast for 3D‐GRE while the vNav images are dominated by T2 contrast due to longer TR and TE. As vNav spatial resolution increases, the magnitude images show: (1) better delineation of anatomical structures, (2) less signal dropout, (3) less geometrical distortion. When acceleration is used, the improvements become more visible around air‐tissue susceptibility interfaces or metal implants due to shorter TE and echo spacing as visible in Figure 3 (see areas indicated by the arrows). Consequently, the ∆B_0_ field maps show better agreement at higher spatial resolution between 3D‐GRE and vNavs maps. Acceleration slightly reduces the signal‐to‐noise ratio as expected from parallel imaging, however the ∆B_0_ field spatial pattern is clearly visible in all protocols. The acquisition parameters listed at the bottom show the rapid ∆B_0_ field mapping capability of vNav32PAT3x2 with acquisition time as low as 242 ms, and effective acceleration factors of up to 5 for vNav96PAT4x2. vNav acceleration enables higher spatial resolution with shorter acquisition time (vNav48PAT4x2 and vNav64PAT4x2) compared to the original unaccelerated vNav (vNav32PAT1) which we demonstrated in prior studies [15, 16].

A voxel‐to‐voxel comparison of ∆B_0_ field maps in an anthropomorphic head phantom and a patient with metal implants is illustrated in Figure 3. For direct comparison, the low‐resolution vNav data are interpolated to match the 3D‐GRE resolution. There is good agreement between 3D‐GRE and vNav field maps in the majority of the brain; however, there are regions of larger difference in areas with high‐susceptibility anisotropy close to the metal implants or skull base. These differences are significantly diminished in the accelerated vNavs. A closer observation of these signal improvements around metal implants can be seen in the magnified images shown in Supplementary Figure S1. The improvement in accelerated vNavs is quantified by the low RMSE and comparable standard deviation values that demonstrate a close agreement with the 3D‐GRE benchmark. In addition, a comparison of ∆B_0_ field mapping for 3 different head poses is shown in Supplementary Figure S2.

**FIGURE 4:**
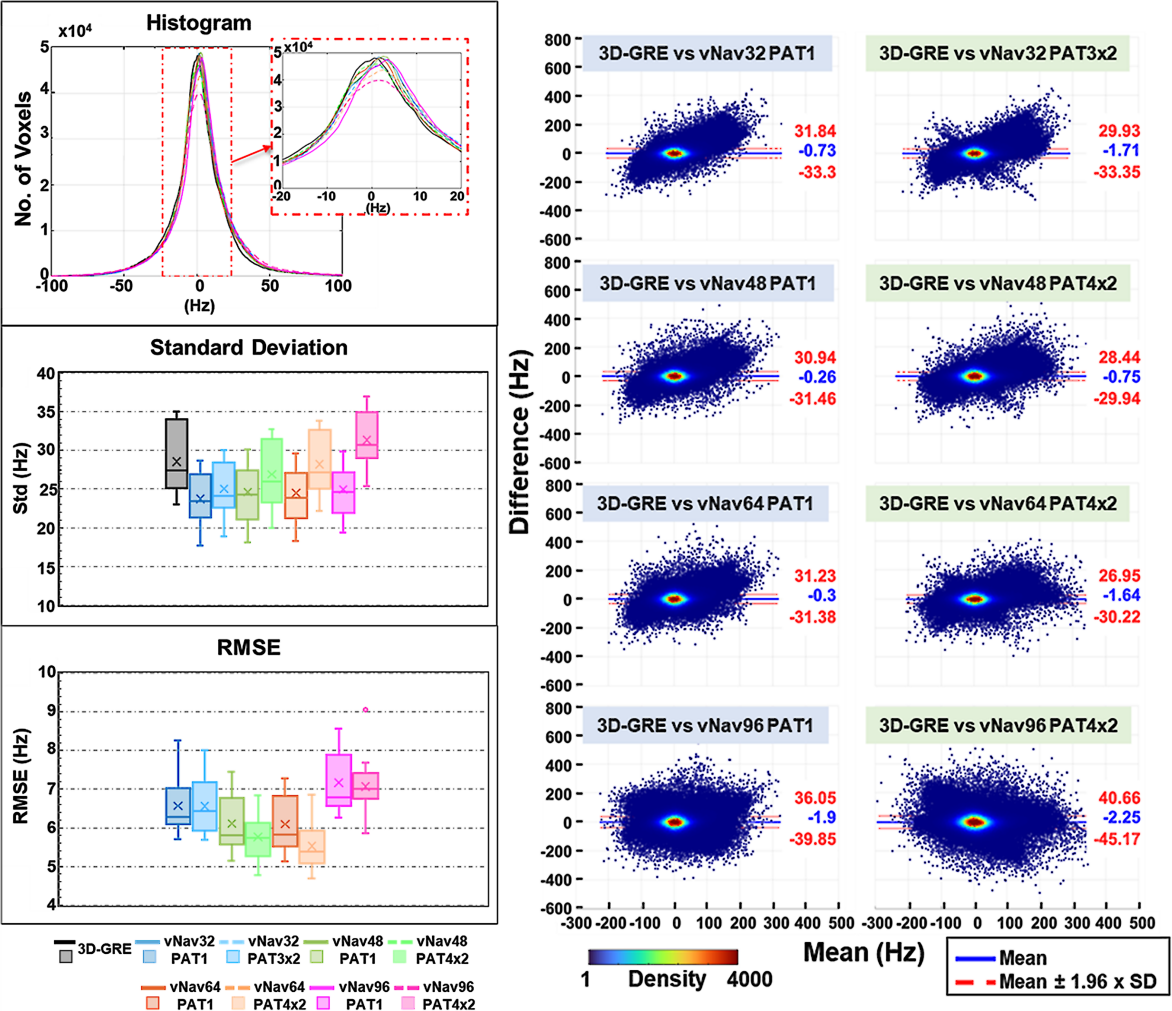
Quantitative analysis of ∆B_0_ field mapping. (A) Histogram, and box plots showing standard deviation and root mean squared error of vNavs with respect to 3D‐GRE. (B) Bland–Altman plots for vNav ∆B_0_ field maps with respect to 3D‐GRE ∆B_0_ field maps. All results are based on pooled data from 11 human participants.

Quantitative analysis performed on all the ∆B_0_ field maps from all 11 subjects is shown in Figure 4. The histograms show very good overlap between the vNav protocols and 3D‐GRE, suggesting a close agreement in the measured ∆B_0_ distribution. The standard deviation is consistently low for unaccelerated vNavs and increases with acceleration, likely due to lower SNR in accelerated vNavs. The standard deviation for the accelerated vNavs was similar to 3D‐GRE. Lower RMSEs are obtained for accelerated vNavs, in particular for vNav64PAT4x2 and vNav48PAT4x2, which seem to indicate a good compromise between spatial resolution, acceleration, and signal‐to‐noise for the fidelity of ∆B_0_ field mapping. Bland–Altman analysis indicates a predominant clustering around the zero‐mean difference, with slightly narrower limits of agreement for accelerated protocols vNav64PAT4x2 and vNav48PAT4x2, which again suggest a sweet spot for the vNavs parameter space. ROI‐based Bland–Altman analysis is shown in Supplementary Figure S3 which shows that most of the differences are driven by voxels in the peripheral regions of the brain. There are smaller standard deviations and good agreement in central brain regions. In central brain regions, a small slope is evident without acceleration (PAT1), and it appears to reduce with acceleration. The anterior part of the outer brain region shows a wider range of ∆B_0_ values compared to the posterior outer region, which is expected due to the effect of frontal and nasal air cavities.

**FIGURE 5:**
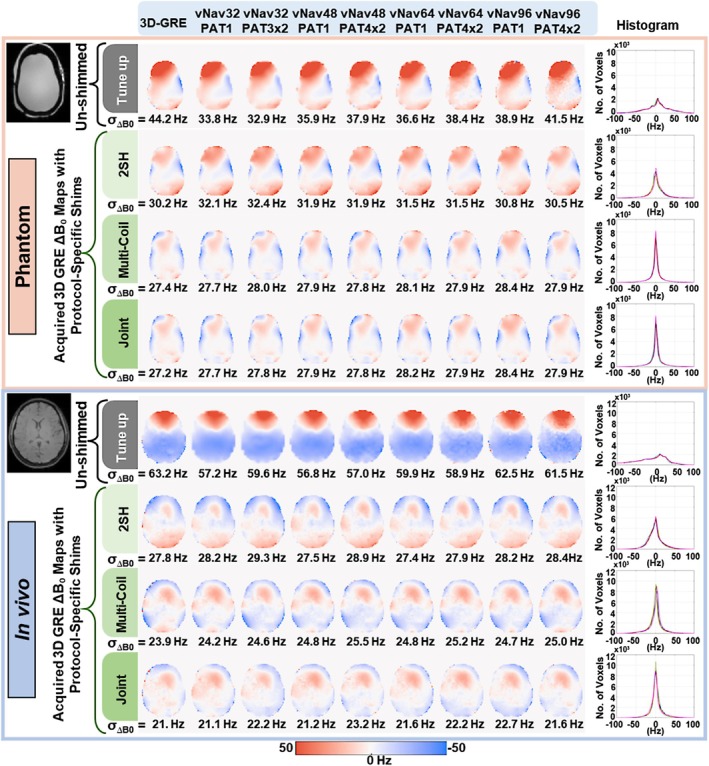
Shimming results comparing ∆B_0_ field maps from an anthropomorphic head phantom and a healthy human volunteer using all ∆B_0_ field maps and three shimming methods. The un‐shimmed ∆B_0_ field maps are acquired with the scanner tune‐up shim for 3D‐GRE and vNav protocols. Optimized ∆B_0_ shimming was computed for spherical harmonic (2SH), 32‐channel multi‐coil shim array (Multi‐coil) and combined 2SH + Multi‐coil (Joint) hardware. Optimized shims were applied to prospectively acquire ∆B_0_ field maps using the same 3D‐GRE protocol for all computations. The corresponding standard deviation for the whole brain volume is shown.

The results obtained with 3D‐GRE and vNav ∆B_0_ field maps for three different ∆B_0_ shimming methods performed in phantom (top) and human (bottom) are shown in Figure 5. Since this experiment did not involve real‐time feedback or motion correction, we were able to apply the full set of 2nd order shim coefficients including the frequency, 1st and 2nd order spherical harmonic terms before starting the measurement without being restricted by shim settling delays. Upon shimming, a marked improvement in ∆B_0_ uniformity compared to the tune‐up shim is obtained for all imaging protocols as shown by the significantly smaller standard deviation and narrower histograms. In addition, there is a gradual progressive improvement in ∆B_0_ uniformity going from (2SH) spherical harmonics (30–23 Hz standard deviation) to 32‐channel AC/DC multi‐coil (24–26 Hz standard deviation) and joint optimization shimming (21–23 Hz standard deviation). The histograms show good agreement between vNavs and 3D‐GRE based shimming for all 3 methods. The experimental results are also supported by simulations shown in Supplementary Figure S4. The shim coefficients and currents computed using the quadratic optimization solver are shown in Supplementary Figure S5. For linear 2SH terms (X, Y, Z), shim currents from all vNav protocols closely align with those derived from 3D‐GRE, even at lower vNav resolutions. For the AC/DC multi‐coil shims, certain channels derived from the lower‐resolution vNav32 protocols exhibited slightly larger deviations from higher‐resolution vNav protocols and 3D‐GRE. These variations likely reflect the AC/DC coils' ability to generate more spatially localized fields, particularly near the periphery of the brain. Nonetheless, the variability in shim currents diminishes with increasing resolution and acceleration, and the overall shimming performance is consistently robust as demonstrated in Figure 5.

**FIGURE 6:**
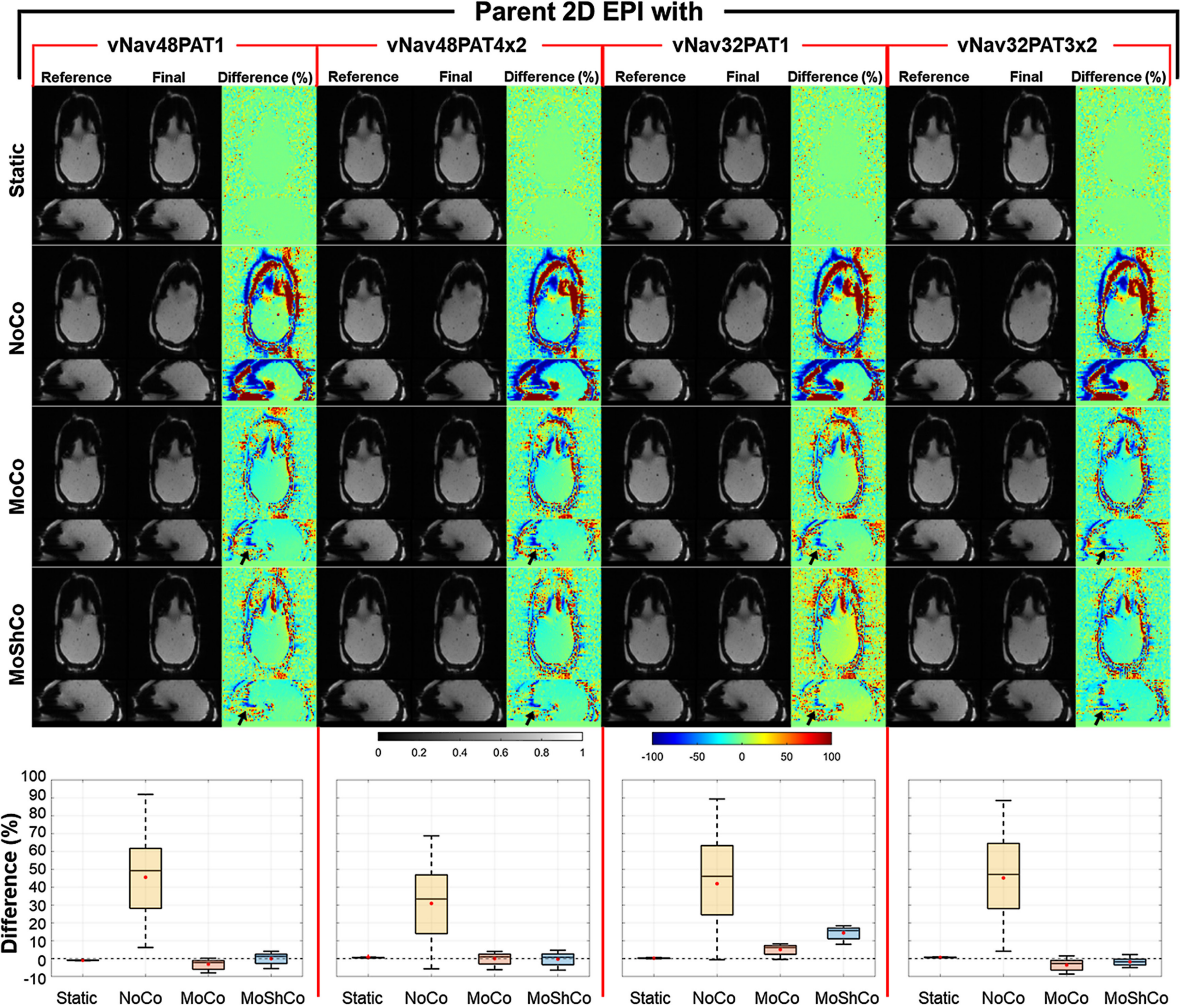
Evaluation of high‐resolution accelerated vNav efficiency for real‐time motion correction in an anthropomorphic head phantom. Top panels show representative 2D‐EPI magnitude images acquired with interleaved vNavs (two resolutions and two acceleration factors) across 30 repeated measurements under four conditions: Static, NoCo (no correction), MoCo (motion correction), and MoShCo (motion and shim correction). Corresponding difference maps depict the relative change between the final (post‐motion) and reference (pre‐motion) images. The phantom was physically rotated and translated consistently for all measurements. Bottom panels show box plots of the percentage difference across the 3D volume for the corresponding condition and vNav configuration. Static shows minimal variability, serving as a baseline. NoCo exhibits significant motion‐induced variation, which is substantially reduced with MoCo and further improved with MoShCo. Red markers indicate mean values; whiskers denote variability.

Figure 6 presents phantom results of motion experiments in an anthropomorphic head phantom with a navigated 2D EPI sequence using four different vNav protocols (vNav48PAT1, vNav48PAT4x2, vNav32PAT1, and vNav32PAT3x2) under four conditions: static, no correction (NoCo), motion correction (MoCo), and motion and shim correction (MoShCo). The reference column represents the initial measurement (before motion), while the final column corresponds to the last measurement (after motion). The relative difference images illustrate the percent relative differences between the final and reference measurements. As expected, large variations are observed in the NoCo condition, indicating the effects of motion. These variations are substantially reduced by MoCo and further minimized around frontal sinus by MoShCo, particularly when using the accelerated navigators. The box plots shown in the bottom panel demonstrates that: (1) the relative difference of the parent 2D EPI images are decreased by the accelerated navigators and (2) the relative difference of the parent 2D EPI images obtained with accelerated navigators is further decreased by MoShCo.

Supplementary Figures S6 and S7 provide additional panels for vNav magnitude and field maps, respectively. The unaccelerated vNav protocols exhibit minimal variations in magnitude images before and after motion correction. However, a slight reduction in SNR and increased aliasing are observed in the final measurements of accelerated vNavs. The motion parameters, displayed in the top panel of Supplementary Figure S8, confirm consistent motion across all protocols and conditions. The bottom panel shows simulated additional shim coefficients based on vNav field maps, highlighting that the static and MoShCo conditions require negligible additional shim corrections, whereas the MoCo condition necessitates additional shim currents to mitigate field inhomogeneities.

**FIGURE 7:**
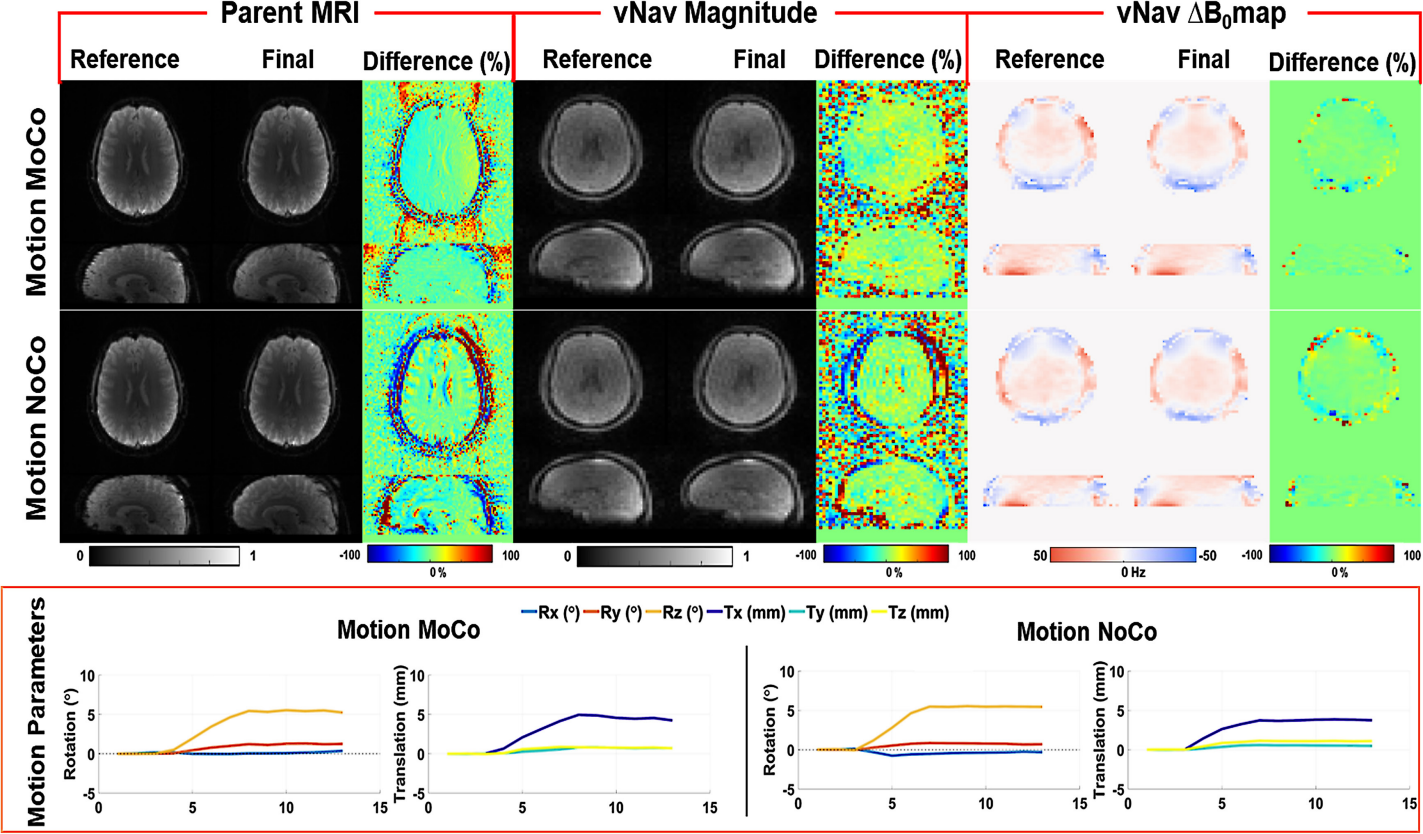
Effect of real‐time motion correction on 2D EPI images using interleaved vNav48PAT4x2 with controlled head movement in a healthy volunteer. The top row shows results with motion correction (Motion MoCo), the middle row shows motion with no correction (Motion NoCo), and motion plots (rotation—Rx,y,z and translation—Tx,y,z) plots are shown at the bottom. Magnitude images and field map relative differences are calculated between the reference (before motion) and final (after motion).

Figure 7 shows the results obtained for motion experiments for 2D EPI with and without real‐time motion correction (MoCo and NoCo) conducted in vivo. Relative difference maps show much larger residual values between the static (reference) and moved images in the case of NoCo compared to MoCo. In the MoCo case, there are residual intensity changes due to receiver coil (B1^−^) differences between the original and moved head positions. The effect of the ACS lines on the GRAPPA reconstruction of accelerated vNav is shown in Supplementary Figure S9, which demonstrates that ACS lines acquired at the start of the experiment are sufficient for robust and high‐quality image reconstruction in the presence of head motion inside the head coil.

**FIGURE 8:**
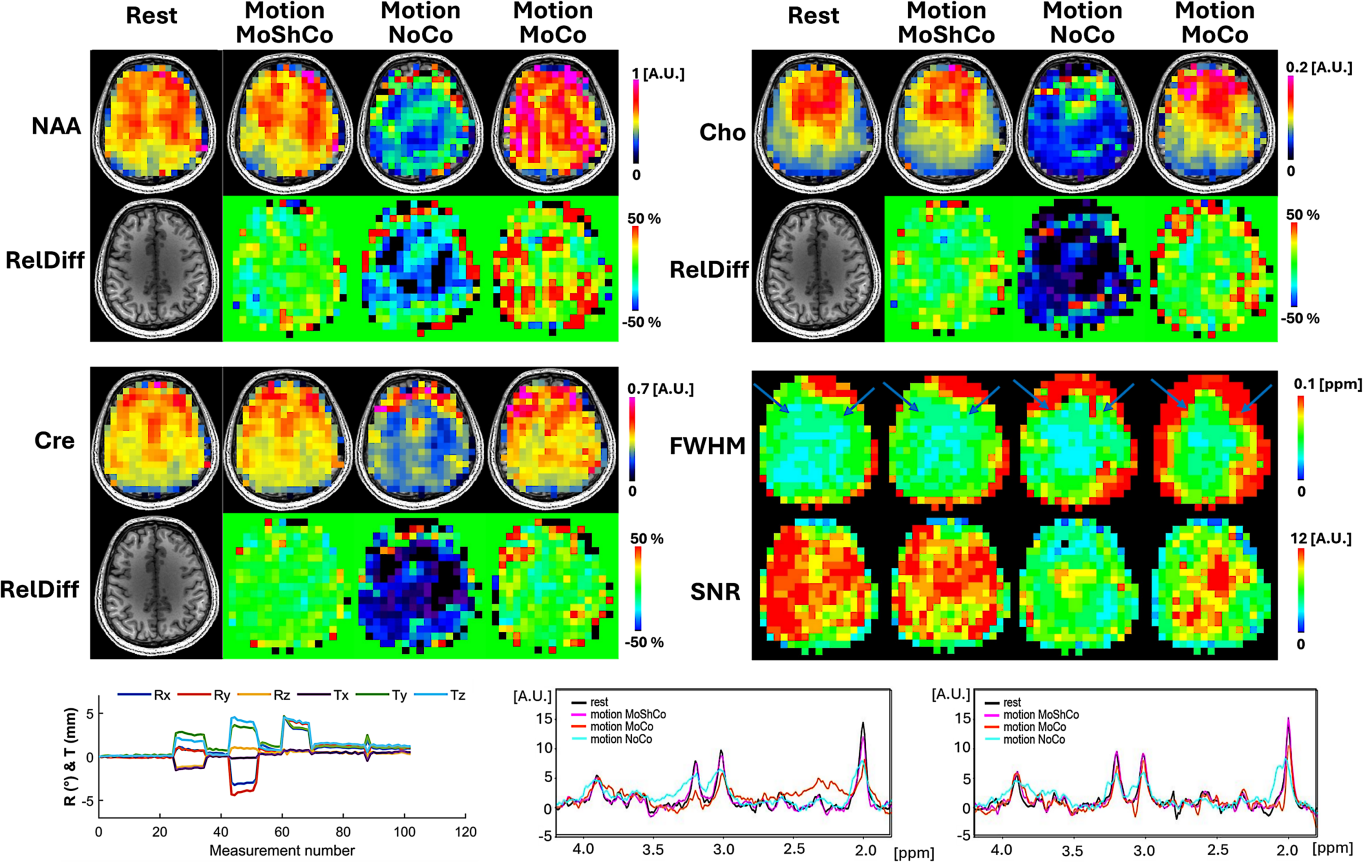
Effect of real‐time motion and shim correction on 3D MRSI using interleaved vNav48PAT4x2 and an AC/DC shim array under controlled head movement in a healthy volunteer. The figure compares conditions: resting (Rest), head movement with real‐time motion and shim correction (Motion MoShCo), head movement without correction (Motion NoCo), and head movement with only motion correction (Motion MoCo). Metabolic maps of total N‐acetyl‐aspartate (NAA), creatine (Cre), and choline (Cho) are presented, along with spectral linewidth (FWHM) and signal‐to‐noise ratio (SNR) maps. Relative differences are calculated between the static maps as reference and the maps of the three motion experiments. At the bottom, spectra from selected voxels (indicated by arrows on FWHM maps) and motion tracking plots from the navigator are displayed.

Figure 8 presents 3D MRSI data obtained under static conditions and during motion with real‐time motion correction (MoCo), motion and shim correction (MoShCo), and no correction (NoCo) using the AC/DC multi‐coil shim array. The highest agreement of metabolic maps, linewidths, and SNR is obtained between static and MoShCo measurements. Motion correction only is insufficient to restore the quality of metabolic maps. This is clearly visible in the spectra from voxels in the frontal brain which show larger peak width and lower peak height for MoCo and NoCo compared to MoShoCo and static experiments. The measurements performed under MoShCo and static conditions provide similar spectra. The vNav ∆B_0_ field maps and motion parameters corresponding to the 3D MRSI acquisition are shown in Supplementary Figure S10. The ∆B_0_ field maps and their relative differences at 5 intervals (before, during and after motion) are illustrated. The static condition shows the least differences followed by the MoShCo condition. These results corroborate with the linewidth and SNR of the MRSI data.

**FIGURE 9:**
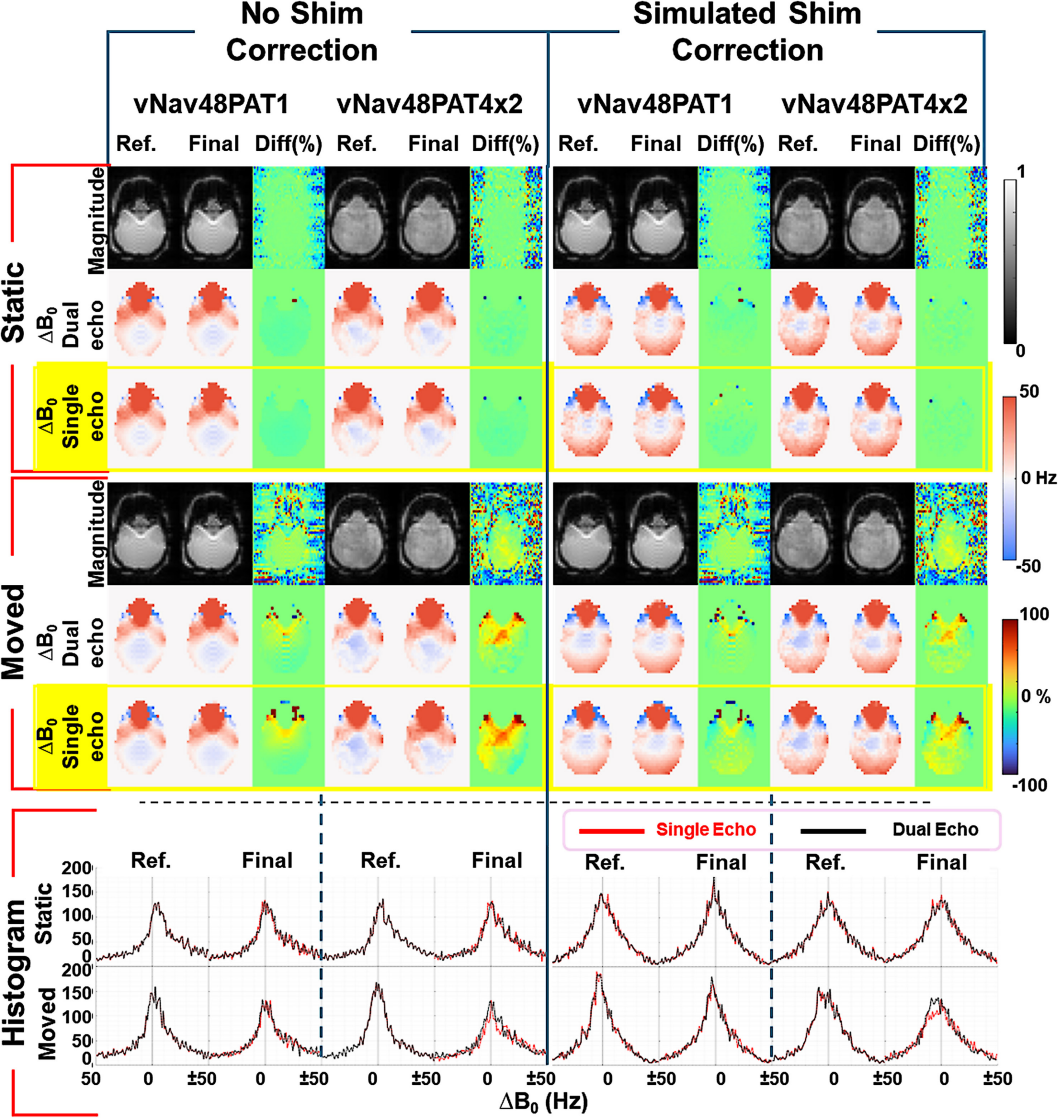
Evaluation of single‐echo ∆B_0_ field mapping under static and chin‐up motion conditions using an anthropomorphic head phantom with vNav48PAT1 and vNav48PAT4x2 navigators. For a representative slice, single‐echo ∆B_0_ field maps are compared to dual‐echo ∆B_0_ field maps for the initial head position (Ref) and the final head position (Final) without doing shim‐update (Left panel) and with the shim‐update (Right panel). Histograms of the bottom show the distribution of ∆B_0_ over the entire 3D brain volume. The motion plots and shimming coefficients are shown in Figure 10.

**FIGURE 10:**
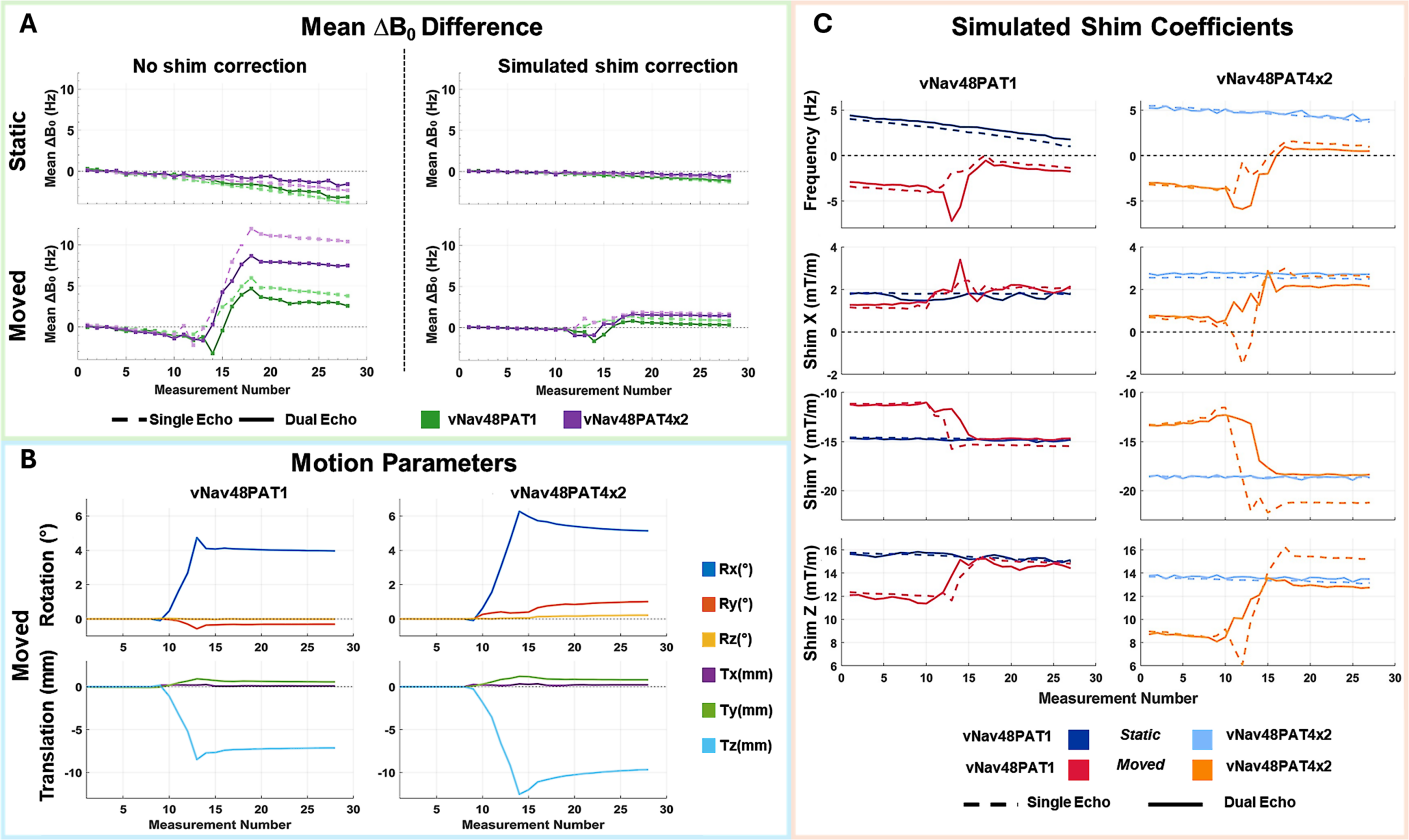
Quantitative evaluation of single‐echo‐derived ΔB₀ field mapping corresponding to the data shown in Figure 9. (A) Mean ΔB₀ difference between single‐echo and dual‐echo field maps across 30 dynamic time‐series measurements for vNav48PAT1 (green) and vNav48PAT4x2 (purple), under static (top) and moved (bottom) conditions, without (left) and with (right) simulated shim correction. (B) Motion parameters estimated from PACE tracking for vNav48PAT1 (left) and vNav48PAT4x2 (right), indicating a ~ 10 mm chin‐up translation (Tz) introduced between measurements 10–15. (C) Simulated shim coefficients (frequency and first‐order X, Y, and Z terms) derived from single‐echo (dashed lines) and dual‐echo (solid lines) ΔB₀ field maps, showing both static (dark blue/light blue) and motion (red/orange) conditions for vNav48PAT1 and vNav48PAT4x2.

Figures 9 and 10 summarize the evaluation of single‐echo‐based vs. dual‐echo ∆B_0_ field mapping in an anthropomorphic head phantom experiment under static and chin‐up motion conditions using unaccelerated (vNav48PAT1) and accelerated (vNav48PAT4x2) navigators. Motion correction was performed in real‐time while shim correction was not enabled to compare the worst‐case scenario for ∆B_0_ field mapping. Figure 9 shows for a representative slice the magnitude images, dual‐echo‐derived ∆B_0_ maps, and retrospectively computed single‐echo ∆B_0_ maps for the reference and final measurements with no‐shim update and with shim update (simulated retrospectively). In the static experiment, the single‐ and dual‐echo ∆B_0_ field maps show good spatial agreement with no noticeable differences between the field maps of the initial head position (Ref) and the final head position (Final). In the motion experiment, there are differences between the initial and final ∆B_0_ field maps when no shim update is performed (Left) as expected, with a similar difference ∆B_0_ field map for both navigators. When the shim update is performed (Right) it can be noticed that the difference between initial and final ∆B_0_ field maps show decreased spatial non‐uniformity with a similar pattern for both navigators. The difference maps confirm that simulated shimming using single‐echo‐derived ∆B_0_ maps closely approximates the dual‐echo results. The histogram plots at the bottom of Figure 9 shows good agreement between single‐echo and dual‐echo ∆B_0_ values over the entire 3D brain volume for all conditions, which further validates this finding.

Figure 10 provides more quantitative analysis of the agreement between single‐echo and dual‐echo navigator ∆B_0_ field mapping. In panel A, the mean ∆B_0_ difference over 30 measurements is plotted for static and motion experiments. Without shim correction (Left), the motion after measurement 10 leads to marked increase in ∆B_0_ offset for both single‐ and dual‐echo methods. After shim correction (Right), the mean ∆B_0_ values return close to baseline, and single‐echo and dual‐echo results remain closely aligned for both vNav protocols. Panel B shows motion parameters estimated using PACE for vNav48PAT1 and vNav48PAT4x2 during motion. A chin‐up translation of approximately 10 mm and a rotation of ~ 5° were introduced between measurements 10–15. Note that slightly large motion was performed during vNav48PAT4x2 experiment. Panel C shows the dynamic frequency and first‐order shim coefficients computed from both single‐echo (dashed) and dual‐echo (solid) ∆B_0_ maps. During the static period at the beginning of the experiment the shim coefficients remain stable across both methods. Following motion both ∆B_0_ field mapping methods estimate a change in frequency and shim terms with similar trends. During the transition measurements (10–15) between the initial and final head position there is a difference between the shim coefficients derived from single‐echo and dual‐echo ∆B_0_ maps as explained further in the discussions. After motion the agreement is regained between shim coefficients of single‐ and dual‐echo ∆B_0_ maps, albeit for the accelerated navigator a small difference between shim coefficients persists also after motion which is further discussed.

## 4 Discussion

The aims of our research were as follows: (1) to implement and evaluate GRAPPA‐accelerated dual‐echo EPI vNavs for accurate mapping and shimming of ∆B_0_ fields and (2) to test GRAPPA‐accelerated dual‐echo EPI vNavs for real‐time motion and shim correction. The motivation for accelerating vNav was to enable shorter scan times and achieve higher spatial resolution with less image deformation and less signal dropout. In addition, the use of GRAPPA‐accelerated single‐echo EPI vNavs for real‐time shim correction was evaluated as well.

Our results indicate that accelerated higher resolution vNavs provide better estimates of the ∆B_0_ field close to tissue‐air interfaces at the edge of the brain and in the proximity of metal implants due to the reduced T2* in the smaller voxels. In addition, the magnitude images obtained by accelerated high resolution vNavs more clearly depict the edge of the brain and skull‐scalp frame, which is important for tracking head position and for brain masking.

The acceleration provides a flexible trade‐off between acquisition time and spatial resolution. Our original unaccelerated low‐resolution vNav was compatible with pulse sequences that had TR longer than 1 s [15, 16]. Using the accelerated navigator, it is possible to simultaneously increase spatial resolution by a factor of 1.5 along each spatial dimension and shorten the acquisition time by a factor of 1.9. The reduced acquisition time of vNav is feasible with more applications enabling TR of 0.5 s or shorter. Our results suggest that a good trade‐off between spatial resolution and acquisition time for high‐quality ∆B_0_ field mapping and head tracking is represented by the GRAPPA 4 × 2 accelerated dual‐echo vNav with 5 mm isotropic resolution and 378 ms acquisition time, or 3.75 mm isotropic resolution and 630 ms acquisition time. For the highest spatial resolution (2.5 mm) an effective acceleration factor of 5 provides good quality field maps and magnitude images; however, the acquisition time of 1302 ms is compatible only with sequences that have long TRs.

Parallel imaging comes with the penalty of higher noise, which becomes more noticeable at the highest spatial resolution and higher acceleration factors due to increased g‐factor noise amplification. However, the unaccelerated vNav protocols have longer echo times, leading to increased signal dropout in regions with large ∆B₀ inhomogeneity compared to accelerated vNavs. Consequently, unaccelerated vNavs may fail to accurately capture voxels with large ∆B₀ offsets, resulting in a smaller standard deviation. The overall impact of noise on ∆B₀ field maps in accelerated vNavs remains smaller than that of signal dropout observed with the unaccelerated vNavs, as indicated in Figure 3 and Supplementary Figure S1. Thus, accelerated vNav protocols show improved agreement with the gold‐standard GRE in shimming performance metrics compared to unaccelerated protocols. Therefore, optimal vNav resolution likely depends on the balance between shim accuracy, motion estimation fidelity, and acceptable acquisition time for the parent sequence. Future studies could evaluate the lower bounds of spatial resolution required for robust shim fitting alone, which may differ from those required for accurate motion tracking.

The result of shimming experiments confirms that the ∆B_0_ field maps derived from accelerated vNavs provide essentially the same quality for whole‐brain static ∆B_0_ shimming as the ∆B_0_ field maps derived from the gold‐standard GRE, while being 1–2 orders of magnitude faster. The Gaussian phase noise amplification due to parallel imaging does not significantly change the underlying spatial pattern of ∆B_0_ field in the brain and hence has minimal impact on the shimming methods which optimize the smoothly varying spatial distribution of the ∆B_0_ field; in other words, the shimming optimizer “sees through” the Gaussian noise. The combination of multi‐coil and spherical harmonic shimming provides the best improvement towards a spatially homogeneous ∆B_0_ field across the whole brain, followed by multi‐coil shimming and spherical harmonic shimming. In particular, advanced multi‐coil shim arrays [33] and high order spherical harmonics [34] hardware have great potential to improve data quality, and our accelerated navigator is fully compatible with such hardware. In real‐time applications, shim settling times significantly influence the performance of real‐time shim update. The long settling times (500–3000 ms) of the scanner's second order shim coils is not compatible with real‐time update; hence only the linear terms of the scanner shims are updated, limiting the ∆B_0_ improvement that can be obtained. In contrast, shim arrays have much faster settling times (1‐2 ms) and provide high‐order shim terms that can be updated in real time, which can improve the ∆B_0_ uniformity better than the linear shim terms. Results obtained with the accelerated navigator for real‐time whole‐brain shimming for 3D MRSI and 2D EPI showed improved data quality during motion.

The accelerated navigator is robust to changes in head position, showing consistent motion tracking and ∆B_0_ field mapping as evidenced by the real‐time motion and shim correction of 2D EPI and 3D MRSI. While large motion may introduce aliasing artifacts and a loss of SNR in the accelerated navigator when the GRAPPA kernel is not updated, as seen in Supplementary Figures S6 and S7, this did not compromise the motion and shim correction of the parent MRI, as evidenced by the results in Figure 6. Because the aliasing artifacts do not cause a change in head shape and position, the rigid registration of PACE accurately estimates motion parameters and corrects the parent sequence. Similarly, the shim optimizer is driven by the smoothly varying spatial pattern of the ∆B_0_ field and can see through the sharp and narrowly localized variation due to aliasing artifacts. Note, when the navigator's pose is not prospectively updated, the motion induced GRAPPA aliasing artifacts are not present. In future implementations, this robustness to aliasing in navigators without prospective pose correction should be weighed against the registration and motion correction advantages of prospectively pose correcting the navigator (see Section 2.2.3).

Correction of continuous head motion can be improved with higher temporal resolution of motion measurement and correction [35]. Shorter vNavs could thus be inserted more frequently to improve correction of continuous head motion. Another effective strategy for vNavs is to selectively reacquire data from periods of continuous head motion [36, 37].

Prediction of motion‐induced ∆B_0_ field changes [3, 32, 38, 39] from single‐echo navigator data is an emerging strategy for accelerating shim update pipelines in real‐time MRI. While our study primarily utilized dual‐echo vNavs for ∆B_0_ field mapping and shimming—given their established accuracy as the gold standard—our analysis in Figures 9 and 10 evaluated the feasibility of a hybrid approach leveraging only the first echo of the dual‐echo acquisition for single‐echo dynamic ∆B_0_ tracking. In an anthropomorphic head phantom experiment under static and chin‐up motion conditions, single‐echo‐derived field maps showed good agreement with dual‐echo maps in both static and motion scenarios. Small deviations were observed during the transition TRs between initial and final head positions because intrinsically the navigator gets updated by PACE with a delay of one TR; hence, the phase image of the single‐echo does not align with the phase reference image measured in the initial TR, while in the case of dual‐echo, both phase images are obtained from the same TR and therefore are always aligned. Furthermore, our experiment was purposely performed under the worst‐case scenario, i.e., the ∆B_0_ field map was not updated in real time by the navigator. The agreement between single‐ and dual‐echo improves with shim update, which indicates that single‐echo field mapping would provide good enough performance for real‐time shim correction. In the case of accelerated navigator, a small difference between single and dual‐echo shim coefficients persisted after motion, which may be partly attributed to reduced SNR and GRAPPA reconstruction instability following motion‐induced misalignment of the coil sensitivity profiles, since no dynamic GRAPPA kernel updates were performed; however, more motion was performed compared to the unaccelerated navigator, which could exacerbate the differences Hence, the single‐echo field mapping may provide an acceptable trade‐off between quality and speed, with an additional two‐fold reduction in navigator acquisition time for a small drop in the accuracy of ∆B_0_ field mapping. Further work is warranted to investigate this trade‐off under various motion, navigator, and parent sequence combinations and assess the potential practical problems related to misalignment, distortion, and artifact differences between the field reference and subsequent single‐echo phase measurements.

Limitations of this work included a longer real‐time feedback delay for the AC/DC shim correction, which was set to 500 ms due to off‐line transfer of ∆B_0_ field map for AC/DC shim calculations. Note that with on‐line ICE integration of the AC/DC shimming, the real‐time feedback delay time can be reduced to the same duration (100 ms) as used for the scanner's 1^st^ order shim update. Importantly, the GRAPPA reconstruction of the accelerated navigator allows a real‐time feedback delay time of 100 ms, which is comparable with what we used in the past [15, 16] for the unaccelerated navigator.

In the MRSI experiments, we acquired a 3D‐encoded 73 mm slab and presented a representative supraventricular slice location, which is generally amenable to linear shimming. A single set of “global” shim currents was optimized for the ∆B_0_ distribution in the entire 73 mm slab. High spatial order ∆B_0_ profiles in inferior slice locations are more effectively nulled with multi‐coil shimming by focusing on thinner slabs, e.g., 10 mm, as done in slice‐by‐slice shimming for EPI [21]. In future work, we aim to assess global multi‐coil shimming performance for MRSI in inferior brain regions when using thicker (~70 mm) 3D‐encoded slabs. Note that even if the initial shimming performance is comparable to 2nd order spherical harmonic shimming, an advantage of multi‐coil shimming is that the shim currents can be rapidly switched to maintain the shimmed ∆B_0_ distribution in the presence of head motion, as demonstrated in Figure 8.

In this study, we evaluated global shimming of a single slab covering the majority of the brain. While whole‐brain shimming is typically performed in fMRI, it has limited effectiveness in regions with severe ∆B₀ inhomogeneities, such as the frontal lobe near the frontal sinus and skull base, where signal dropout is prevalent. Improved ∆B₀ homogeneity in these areas requires slice‐by‐slice shimming with high‐order shim terms to account for the complex spatial distribution of field inhomogeneities. Dynamic or slice‐optimized shimming of 2D multi‐slice EPI [40] for fMRI applications has been demonstrated with the AC/DC shim array [27], which improves ∆B_0_ nulling performance by focusing on localized ∆B_0_ inhomogeneity on a slice‐by‐slice basis. By acquiring ∆B₀ maps using vNavs each TR, shim settings can be dynamically updated to correct for ∆B₀ changes induced by head motion [41] or respiration [42]. Accelerated vNavs and faster computational pipelines are important for fMRI where short TRs (1–2 s) are common. Shim arrays, with their ability to generate complex ∆B₀ field patterns with rapid switching times, are well suited for real‐time slice‐by‐slice shim updates to enhance fMRI data quality.

In future work, we will test the efficacy of vNavs for dynamic, slice‐optimized, real‐time shim updating and integrate single‐echo based vNav motion and shim correction into the online pipeline. The utility of vNavs for shim updating and/or retrospective distortion correction could also be compared to alternative approaches such as the EPI phase‐based ∆B_0_ tracking approach for time‐series EPI data [43].

## 5 Conclusion

In summary, GRAPPA acceleration enables high spatial resolution and short acquisition time EPI volume navigators, providing robust high‐quality magnitude images with dual‐ and single‐echo ∆B_0_ field maps that can be used for real‐time motion correction and shim update. The fast acquisition provides flexibility and makes feasible the combination of accelerated volume navigator with many application sequences. It is expected that the use of accelerated navigators will significantly improve data quality in applications which are critically dependent on scan stability and ∆B_0_ field uniformity.

## Author Contributions

N.B.J, J. S, R. F, N. A, O.C.A: method development, study design, data measurement, clinical translation, and data analysis; Y. C and A.v.K: method development. All authors participated in manuscript writing and editing.

## Supporting information


**Data S1:** Supplementary information.

**Figure S1:** Comparison of magnitude images supporting Figure 3, highlighting signal variations in a subject with metal implants. The red arrow indicates signal loss observed in unaccelerated vNavs, while the green arrow shows signal gain achieved in accelerated vNavs and 3D‐GRE due to shorter TE. These improvements demonstrate the advantage of acceleration in reducing signal dropout, particularly near regions affected by susceptibility‐induced distortions.

**Figure S2:** Comparison of ∆B_0_ field maps from a healthy human volunteer for three head poses. Field maps are acquired with tune‐up shim and no shim adjustments were performed between poses. Right–left head rotation of ±25° is compared to the neutral head position.

**Figure S3:** Bland–Altman plots comparing ΔB_0_ field maps for different brain regions of interest derived from vNav protocols to the gold‐standard 3D‐GRE method, supporting Figure 4. The brain was derived in four regions of interests using a combination of central, peripheral, anterior, and posterior regions.

**Figure S4:** Simulations of shimmed ∆B_0_ field maps in the anthropomorphic head phantom and a healthy human volunteer. The un‐shimmed ∆B_0_ field maps were measured with the scanner tune‐up shim for 3D‐GRE and vNav protocols. The un‐shimmed ∆B_0_ field maps were used as input for simulations of shimmed ∆B_0_ field maps assuming three hardware configurations: spherical harmonic (2SH), 32‐channel multi‐coil shim array (Multi‐coil), and combined 2SH + Multi‐coil (Joint). Compensatory shimming fields were applied retrospectively to the un‐shimmed 3D‐GRE ∆B_0_ field map for all computations.

**Figure S5:** Comparison of shim coefficients and currents for 2SH, ACDC multi‐coil and joint shimming methods for phantom and in vivo dynamic shimming shown in Figure 5. The solid black line corresponds to gold‐standard 3D GRE and the markers indicate the vNavs shim currents. Shim currents derived from ∆B_0_ field maps acquired with vNavs of higher spatial resolution and acceleration tend to be closer to the shim currents obtained from 3D GRE.

**Figure S6:** Comparison of magnitude images of vNavs (2 resolutions and 2 accelerations) are shown for static, NoCo, MoCo, and MoShCo conditions, acquired during the experiments shown in Figure 6. The relative difference maps indicate the relative difference of the final measurement (after motion) with respect to the reference measurement (before motion).

**Figure S7:** Comparison of ∆B_0_ field maps of vNavs (2 resolutions and 2 accelerations) are shown for static, NoCo, MoCo, and MoShCo conditions, acquired during the experiments shown in Figure 6. The relative difference maps indicate the relative difference of the final measurement (after motion) with respect to the reference measurement (before motion).

**Figure S8:** Comparison of shim currents calculated for 4 vNavs (2 resolutions and 2 accelerations) during the motion experiments of Figure 6. The motion parameter plots (top) show similar motion through all conditions and vNav protocols. The phantom was rotated ~15° and translated ~10 mm along the *z* direction. The calculated shim currents (bottom) for the static and MoShCo conditions show that there were no additional shim currents required post data acquisition. By contrast, the shim currents required for MoCo are significantly larger. In addition, large shim current changes are observed during the transition between different head poses for all experiments.

**Figure S9:** Evaluation of the ACS lines for GRAPPA reconstruction of accelerated vNav images during motion. Comparison between static and motion vNav images with and without re‐acquisition of ACS lines. ACS lines were re‐acquired only for the single‐frame (static) navigator protocol (setter protocol) in the initial and final head positions. In the case of the multi‐frame (dynamic) navigator protocol which is used for real‐time motion correction the ACS lines were acquired only in the initial head position (initial ACS). It can be seen that the magnitude images and ∆B_0_ field maps obtained by the navigator in the final head position with the initial ACS lines are free of artifacts and have the same appearance as the those obtained with the final ACS lines. Note that the CSF contrast of ventricles are different in the setter versus the navigator protocols due to magnetization history (one TR versus multiple TRs). The real‐time update of the navigator localization was not enabled in this experiment purposely to see the effects of ACS reacquisition. The results with real‐time update of navigator localization are shown in Figure 7 (see the navigator images before and after the head motion reconstructed with the initial ACS lines).

**Figure S10:** Comparison of vNav ΔB_0_ field maps acquired interleaved during in vivo MRSI measurements under static, MoCo, and MoShCo conditions, corresponding to the conditions shown in Figure 8. Representative ΔB_0_ field maps from five blocks, with the numbers above each block indicating the corresponding motion events, as shown in the motion parameter plots. The top row displays vNav ΔB_0_ maps for one measurement from each block, while the middle row shows the relative difference maps computed relative to block 1 (before motion). The bottom row depicts motion parameter plots, showing rotational (Rx, Ry, Rz) and translational (Tx, Ty, Tz) displacements across the measurement time course.

## Data Availability

The data that support the findings of this study are available from the corresponding author upon reasonable request.
