# Supplementary material for: Accelerated Navigator for Rapid ∆B0 Field Mapping for Real‐Time Shimming and Motion Correction of Human Brain MRI
Source: NMR Biomed. 2025 Sep 4;38(10):e70126. doi: 10.1002/nbm.70126 (PMC12409692; doi:10.1002/nbm.70126)
Supplement: Supplementary file 1 — Data S1: Supplementary information. [file NBM-38-e70126-s008.docx]

**Supplementary Materials**

**to**

***“Accelerated navigator for rapid ∆B_0_ field mapping for real-time shimming and motion correction of human brain MRI”***

**
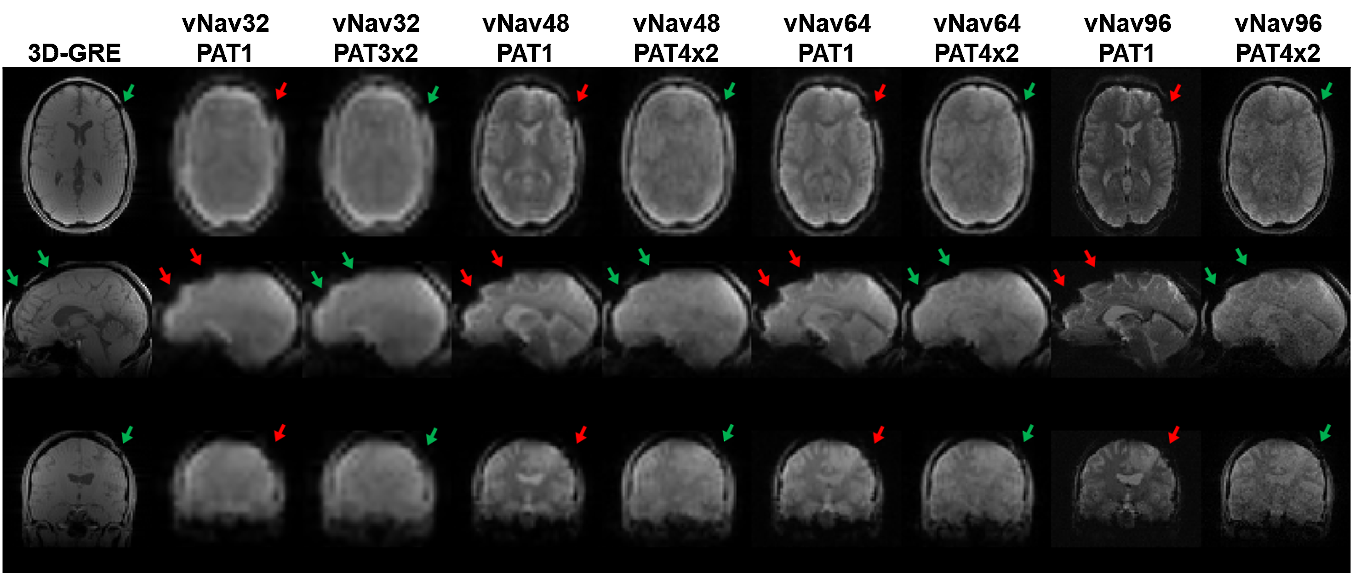
**

**Supplementary Figure S1.** Comparison of magnitude images supporting Figure 3, highlighting signal variations in a subject with metal implants. The red arrow indicates signal loss observed in unaccelerated vNavs, while the green arrow shows signal gain achieved in accelerated vNavs and 3D-GRE due to shorter TE. These improvements demonstrate the advantage of acceleration in reducing signal dropout, particularly near regions affected by susceptibility-induced distortions.


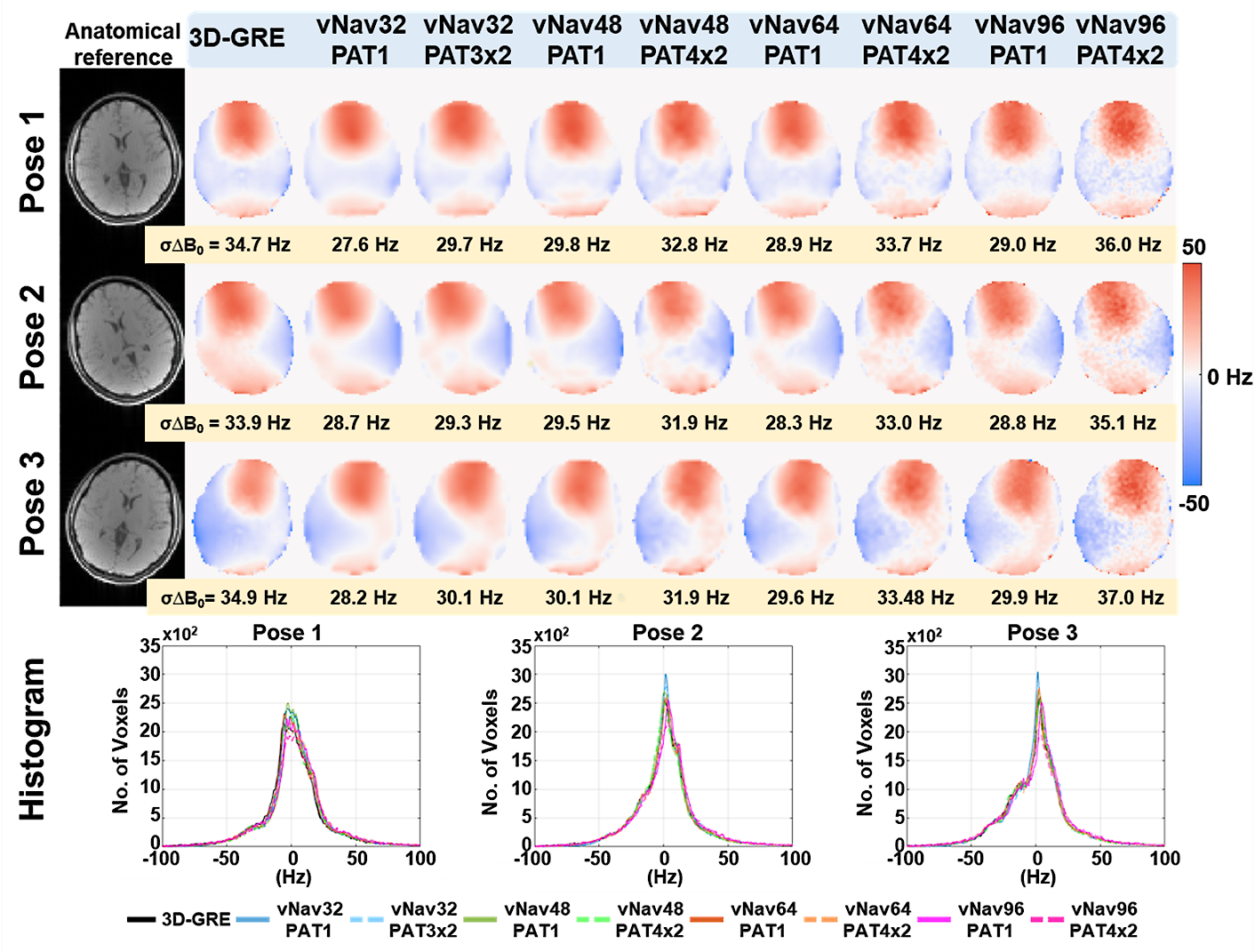


**Supplementary Figure S2.** Comparison of ∆B_0_ field maps from a healthy human volunteer for three head poses. Field maps are acquired with tune-up shim and no shim adjustments were performed between poses. Right-left head rotation of ±25° is compared to the neutral head position.


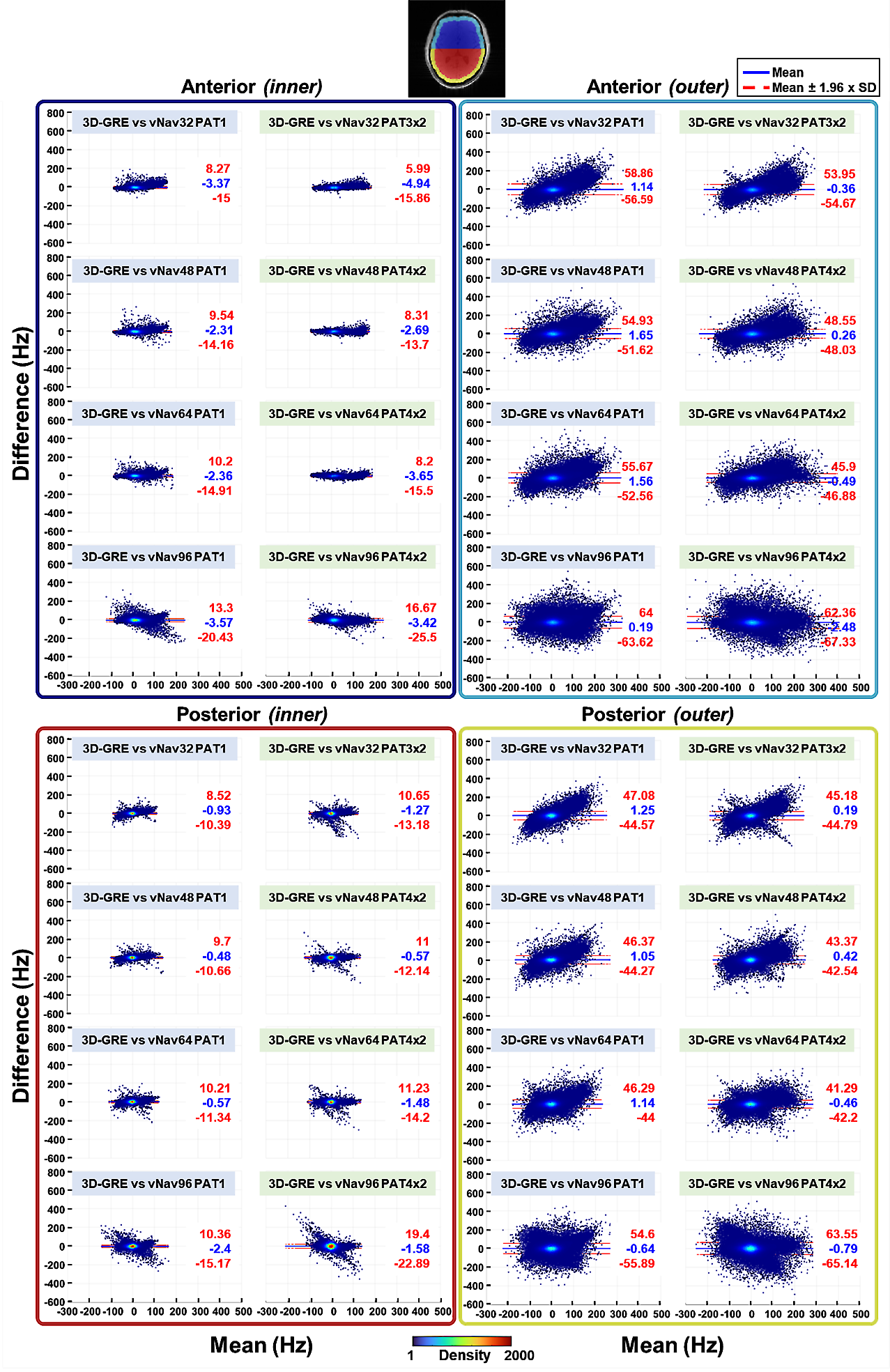


**Supplementary Figure S3.** Bland-Altman plots comparing ΔB_0_ field maps derived from vNav protocols with the gold-standard 3D-GRE method, in support of Figure 4. Each panel represents a specific vNav protocol and shows the agreement with 3D-GRE across various acceleration (PAT) settings. The figure is divided into four regions: anterior-inner, anterior-outer, posterior-inner, posterior-outer, based on brain masks overlaid on the anatomical image.


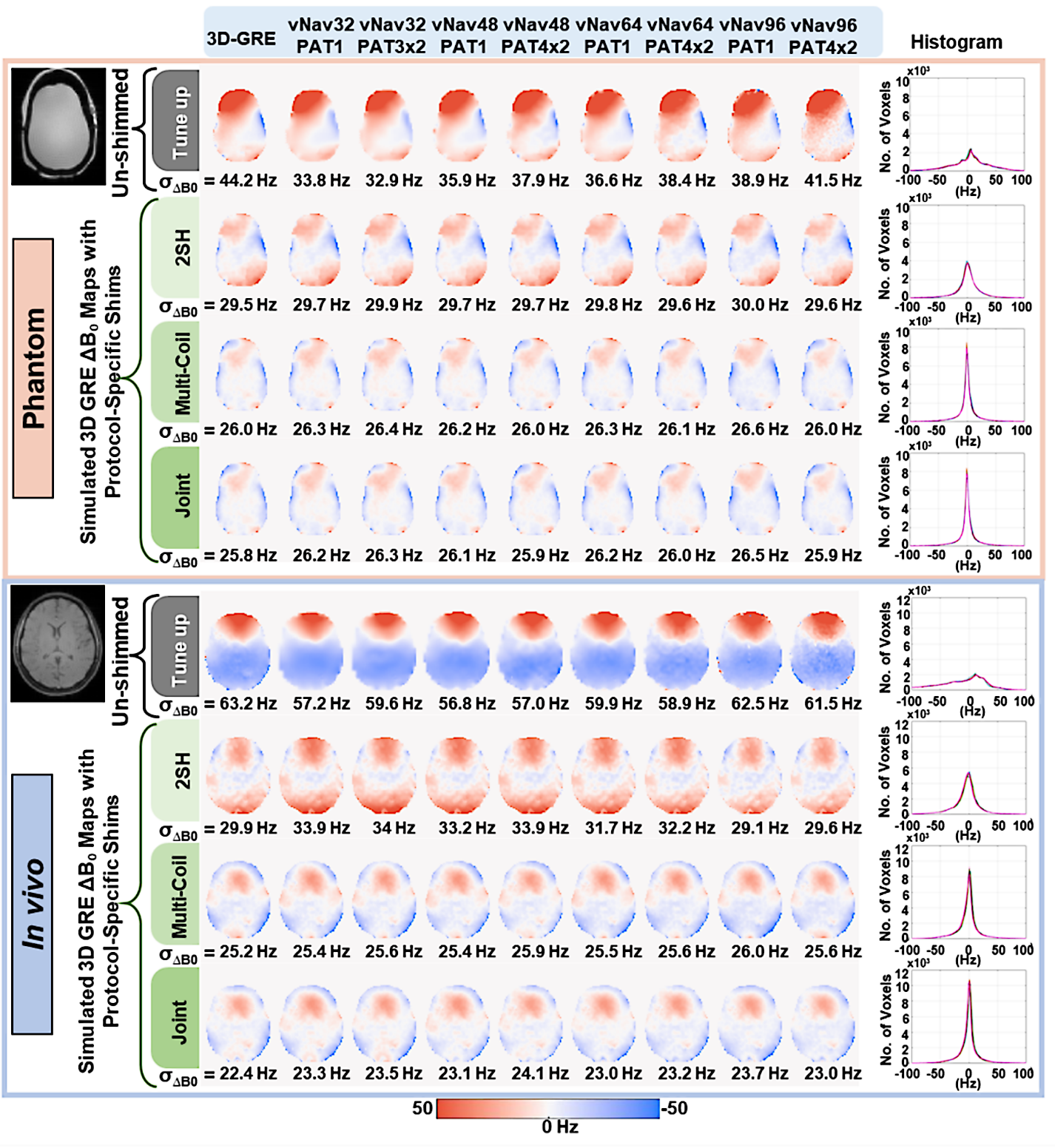
**Supplementary Figure S4.** Simulations of shimmed ∆B_0_ field maps in the anthropomorphic head phantom and a healthy human volunteer. The un-shimmed ∆B_0_ field maps were measured with the scanner tune-up shim for 3D-GRE and vNav protocols. The un-shimmed ∆B_0_ field maps were used as input for simulations of shimmed ∆B_0_ field maps assuming three hardware configurations: spherical harmonic (2SH), 32-channel multi-coil shim array (Multi-coil) and combined 2SH+Multi-coil (Joint). Compensatory shimming fields were applied retrospectively to the un-shimmed 3D-GRE ∆B_0_ field map for all computations.


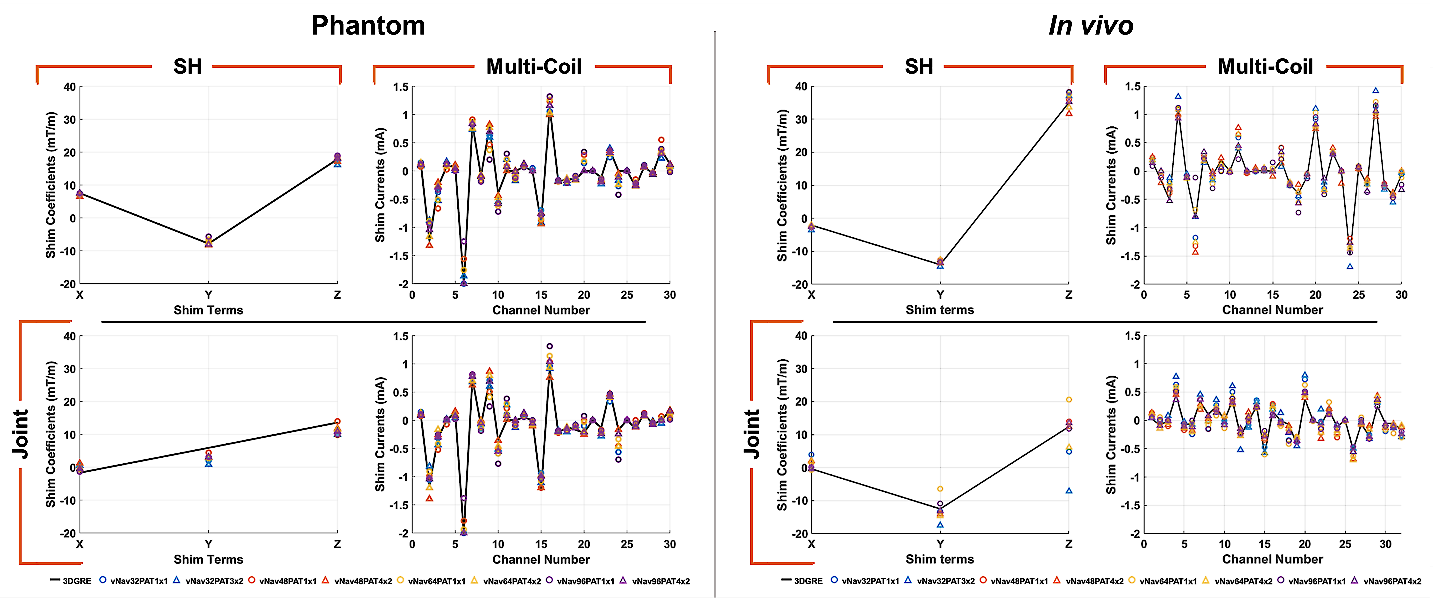


**Supplementary Figure S5.** Comparison of shim coefficients and currents for 2SH (only linear shim coefficients shown), ACDC multi-coil and joint shimming methods for phantom and in vivo dynamic shimming shown in Figure 5. The solid black line corresponds to gold-standard 3D GRE and the markers indicate the vNavs shim currents. Shim currents derived from ∆B_0_ field maps acquired with vNavs of higher spatial resolution and acceleration tend to be closer to the shim currents obtained from 3D GRE.


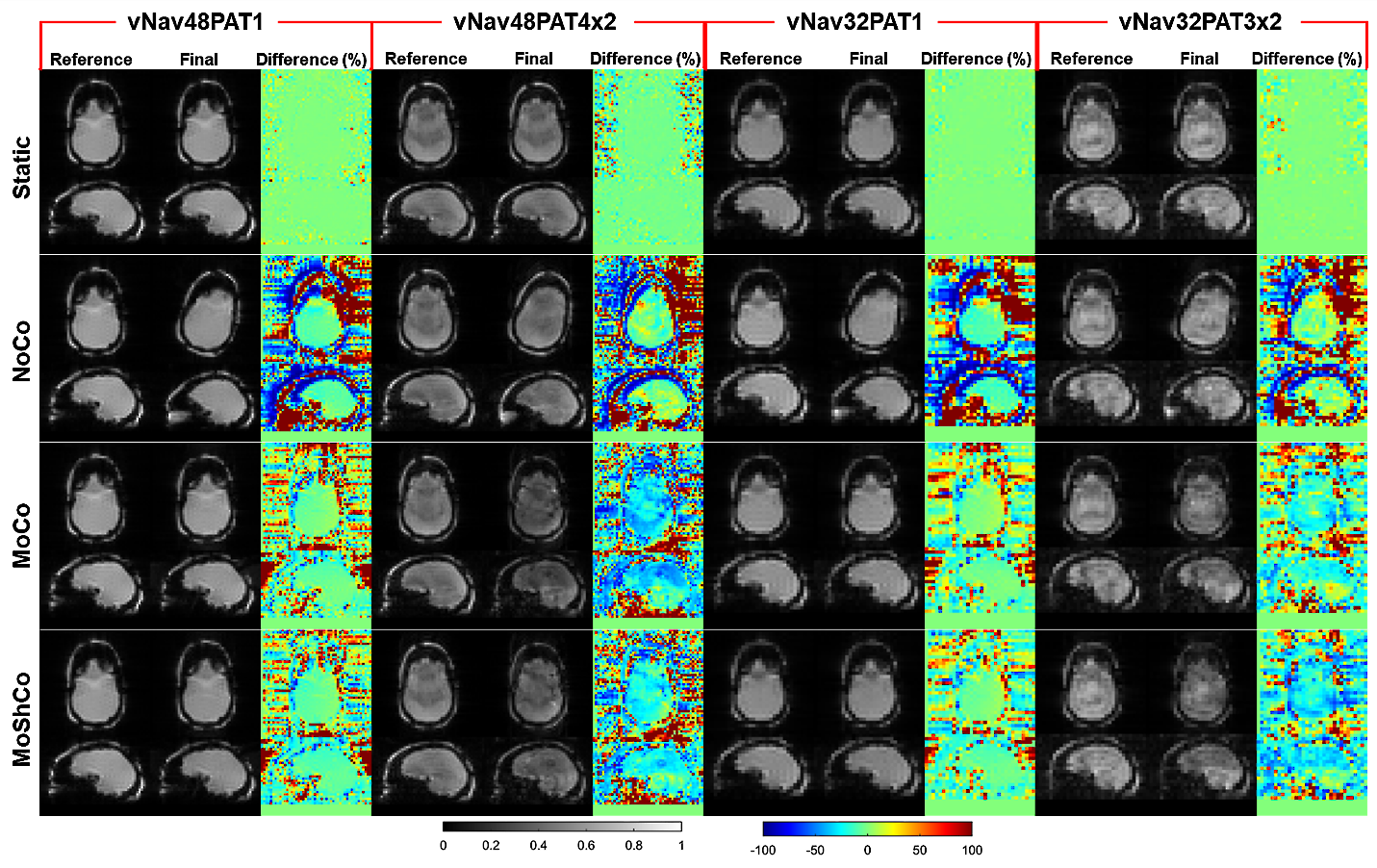


**Supplementary Figure S6.** Comparison of magnitude images of vNavs (2 resolutions and 2 accelerations) are shown for static, NoCo, MoCo and MoShCo conditions, acquired during the experiments shown in Figure 6. The relative difference maps indicate the relative difference of the final measurement (after motion) with respect to the reference measurement (before motion).


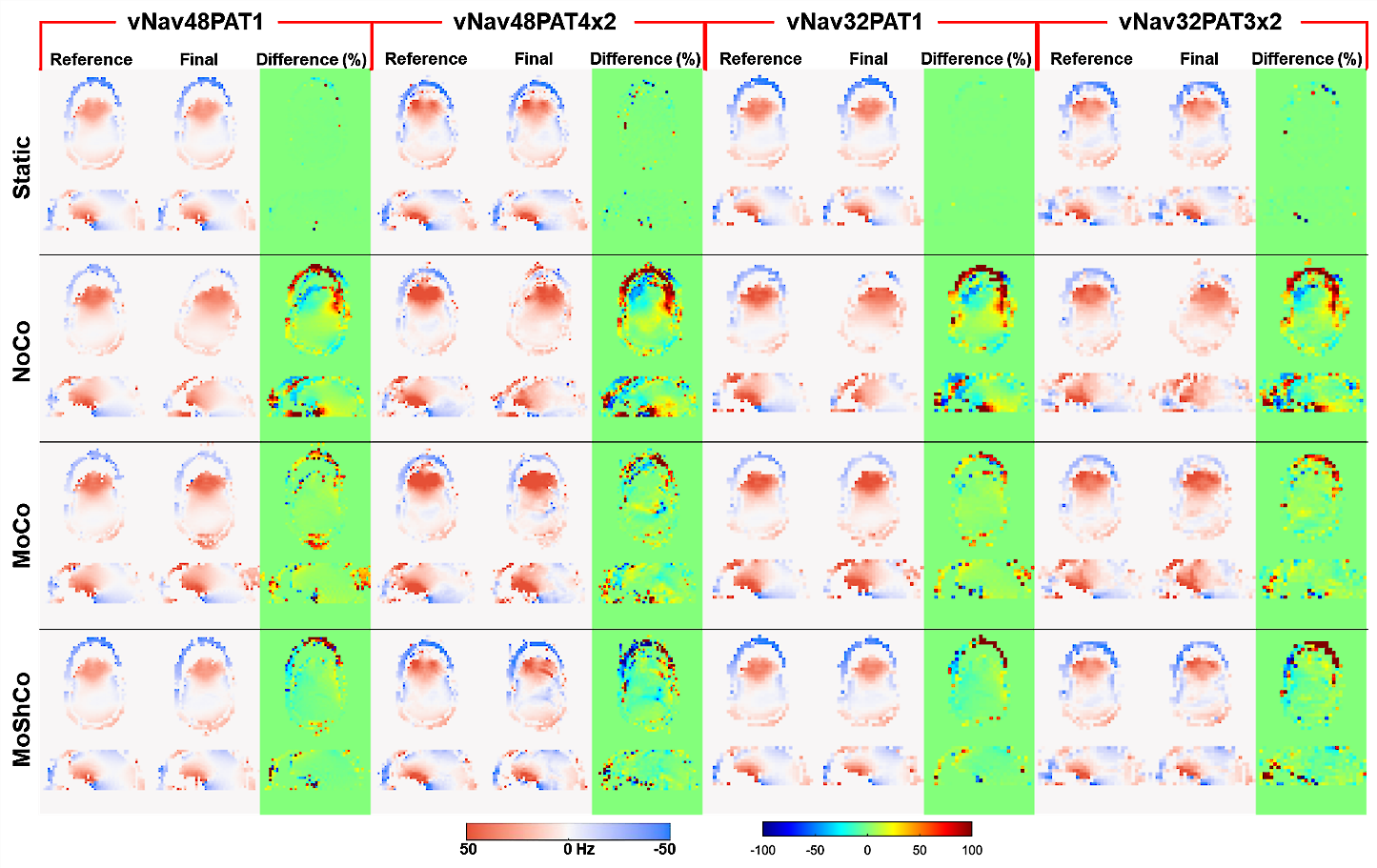


**Supplementary Figure S7.** Comparison of ΔB_0_ field maps of vNavs (2 resolutions and 2 accelerations) are shows for static, NoCo, MoCo and MoShCo conditions, acquired during the experiments shown in Figure 6. The relative difference maps indicate the relative difference of the final measurement (after motion) with respect to the reference measurement (before motion).


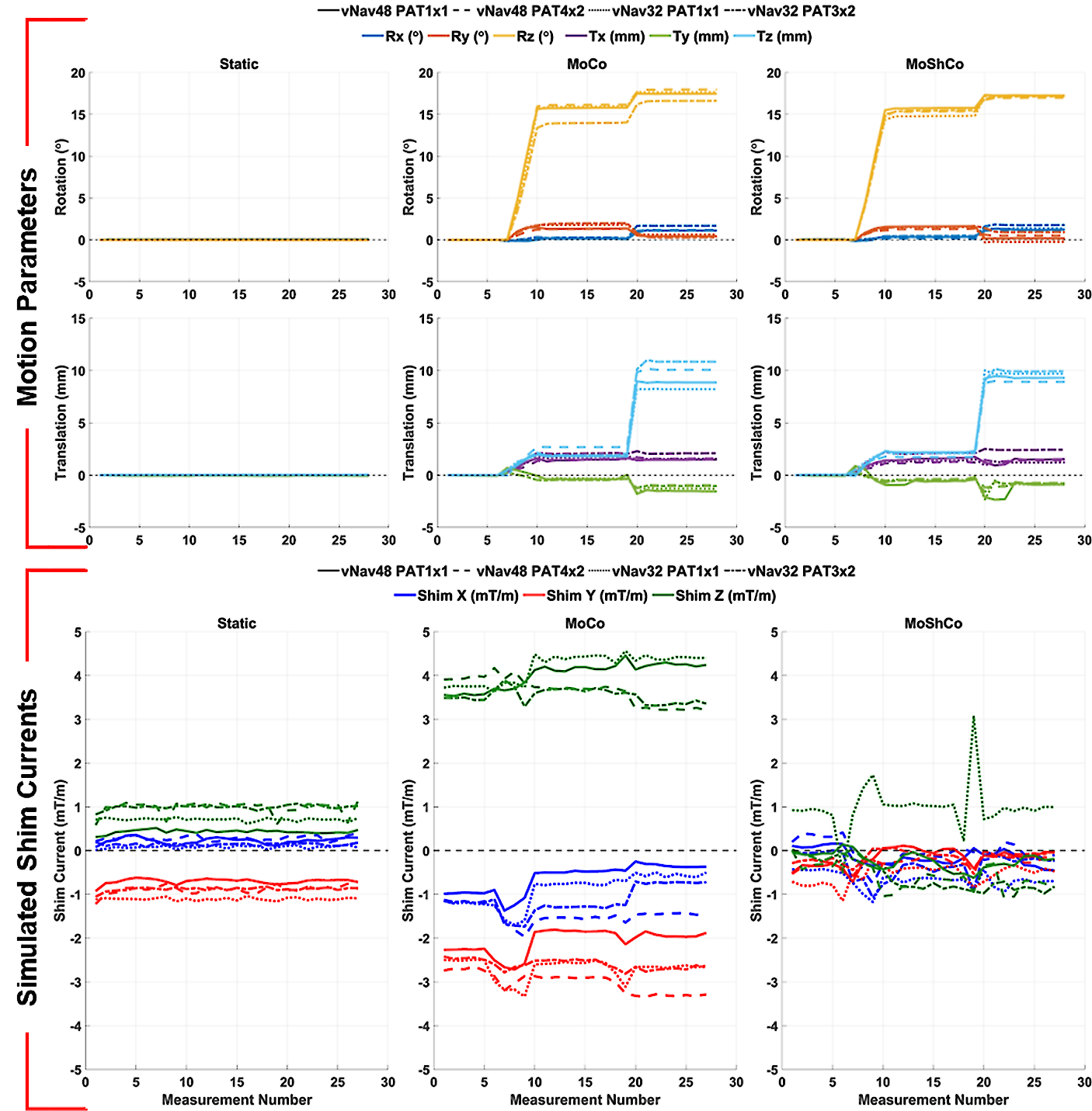


**Supplementary Figure S8.** Comparison of shim currents calculated for 4 vNavs (2 resolutions and 2 accelerations) during the motion experiments of Figure 6. The motion parameter plots (top) show similar motion through all conditions and vNav protocols. The phantom was rotated ~15 degrees and translated ~ 10 mm along the z direction. The calculated shim currents (bottom) for the Static and MoShCo conditions show that there were no additional shim currents required post data acquisition. By contrast the shim currents required for MoCo are significantly larger. In addition, large shim current changes are observed during the transition between different head poses for all experiments.


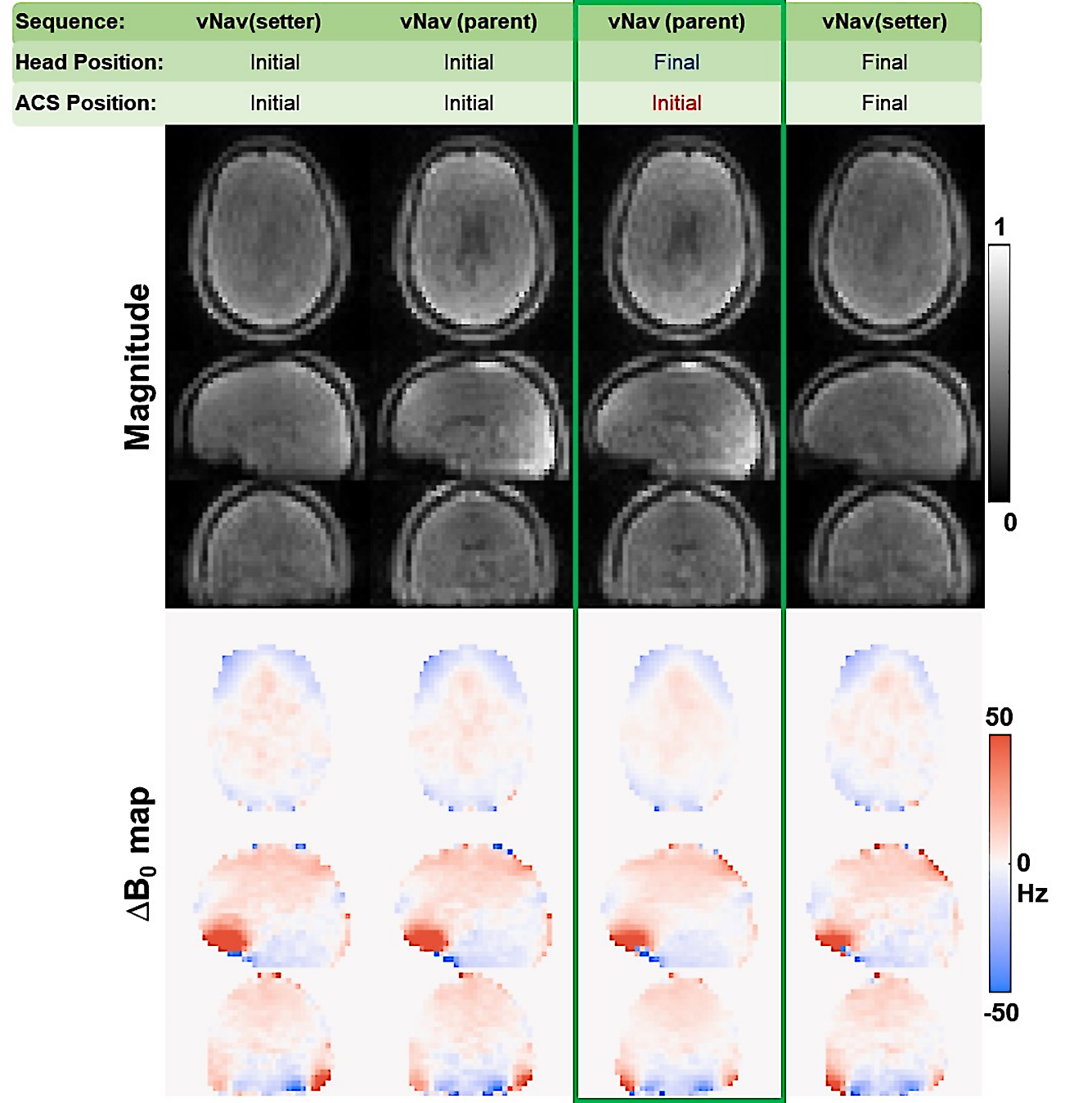


**Supplementary Figure S9.** Evaluation of the ACS lines for GRAPPA reconstruction of accelerated vNav images during motion. Comparison between static and motion vNav images with and without re-acquisition of ACS lines. ACS lines were re-acquired only for the single-frame (static) navigator protocol (setter protocol) in the initial and final head positions. In the case of the multi-frame (dynamic) navigator protocol which is used for real-time motion correction the ACS lines were acquired only in the initial head position (initial ACS). It can be seen that the magnitude images and ∆B_0_ field maps obtained by the navigator in the final head position with the initial ACS lines are free of artifacts and have the same appearance as the those obtained with the final ACS lines. Note, that the CSF contrast of ventricles are different in the setter versus the navigator protocols due to magnetization history (one TR versus multiple TRs). The real-time update of the navigator localization was not enabled in this experiment purposely to see the effects of ACS reacquisition. The results with real-time update of navigator localization are shown in Figure 7 (see the navigator images before and after the head motion reconstructed with the initial ACS lines).


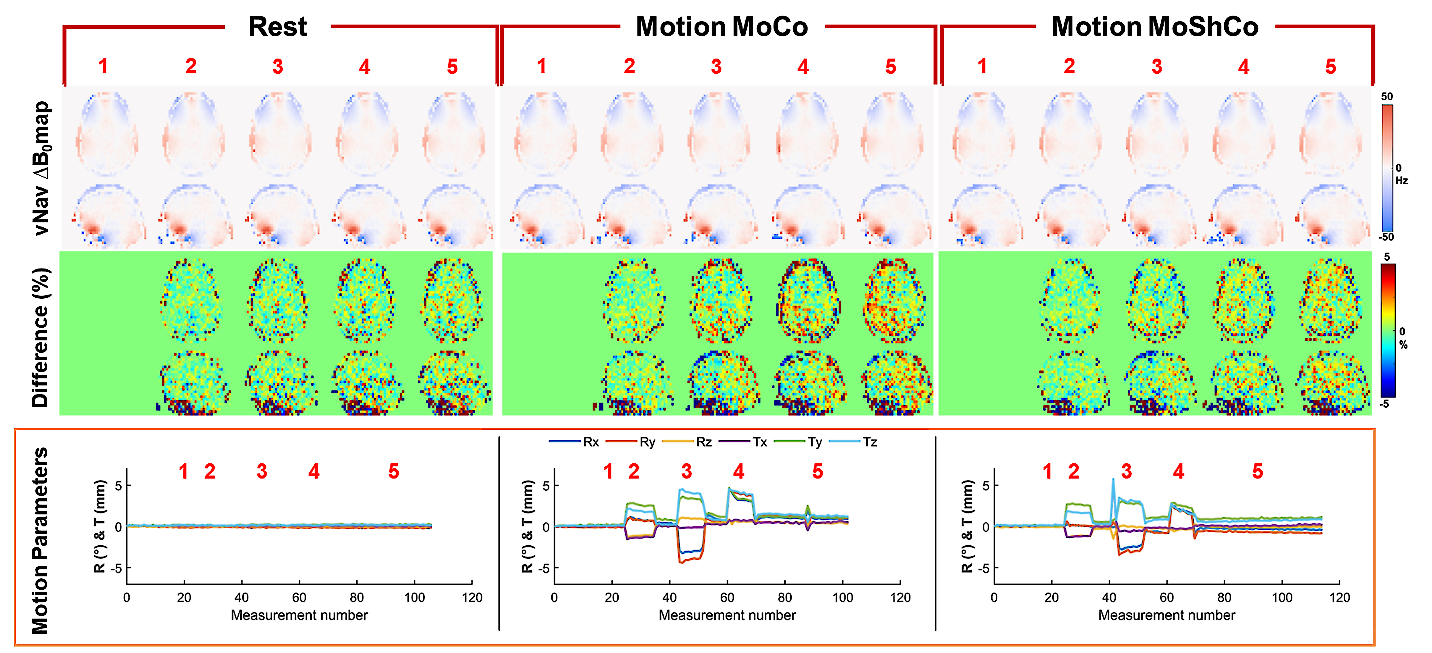


**Supplementary Figure S10.** Comparison of vNav ΔB_0_ field maps acquired interleaved during vivo MRSI measurements under static, MoCo, and MoShCo conditions, corresponding to the conditions shown in Figure 8. Representative ΔB_0_ fieldmaps from five blocks, with the numbers above each block indicating the corresponding motion events, as shown in the motion parameter plots. The top row displays vNav ΔB_0_ maps for one measurement from each block, while the middle row shows the relative difference maps computed relative to block 1 (before motion). The bottom row depicts motion parameter plots, showing rotational (Rx, Ry, Rz) and translational (Tx, Ty, Tz) displacements across the measurement time course.
